# Preparation and Investigation of Solvatomorphs of Anti-Tumor Candidate RA-XII

**DOI:** 10.3390/pharmaceutics18101285

**Published:** 2026-10-10

**Authors:** Feng Cao, Song Zhang, Qicheng Duan, Haitao Dou, Yinghui Liang, Ninghua Tan

**Affiliations:** Department of TCMs Pharmaceuticals, School of Traditional Chinese Pharmacy, China Pharmaceutical University, Nanjing 211198, China; 18435166854@163.com (F.C.); z2363290915@163.com (S.Z.); qc_duan@163.com (Q.D.); shandongdouhaitao@126.com (H.D.); yhliang2025@163.com (Y.L.)

**Keywords:** RA-XII, solvatomorphs, single crystals, hydrate, molecular dynamic simulations

## Abstract

RA-XII, a unique natural cyclopeptide, is a potential anti-colorectal cancer candidate by mainly inhibiting protective autophagy, SREBP-1-dependent lipid biogenesis and suppressing activation of the Akt-mTOR pathway in HCT-116 cells. However, there have been no investigations into its solid forms, and its poor solubility restricts its application in the development of clinical anticancer agents. **Background/Objectives**: To investigate and develop advantageous medicinal solid forms of RA-XII and further improve its solubility characteristics. **Methods**: Various recrystallization methods were used to screen crystal forms of RA-XII, and single crystals (Forms I, II and V) were obtained by the vapor diffusion method. The forms were characterized by powder X-ray diffraction (PXRD), spectroscopy (FTIR, Raman), thermal analyses (DSC, TGA) and scanning electron microscopy (SEM). Structural parameters were determined by single crystal X-ray diffraction (SCXRD). The solubility and intrinsic dissolution rate were determined in a pH 7.4 pharmaceutical buffer solution with water. Hirshfeld surface analyses, molecular electrostatic potential surface (MEPs) analyses and molecular dynamics (MD) simulations were employed to reveal differences in hydrogen bond interactions of the crystal structure. **Results**: Five solvatomorphs and an amorphous form of RA-XII were successfully obtained. Then, three single crystals of Forms I, II and V were obtained, and their conformations were investigated. Hirshfeld surface analyses and crystal structures revealed significant differences in hydrogen bonding interactions and molecular packing. Moreover, MEPs analyses and MD suggested the formation mechanisms of RA-XII solvatomorphs, in which the hydrogen bonding strengths increase between RA-XII and solvent molecules. **Conclusions**: Form I is physically more stable and exhibits great solubility in water. Form I possesses significant medicinal advantages. These findings provide valuable insights into understanding the solvatomorph mechanisms and the quality control of RA-XII.

## 1. Introduction

The solid properties of active pharmaceutical ingredients (APIs) are essential for pharmaceutical research, as these properties can significantly affect the performance of solid pharmaceutical formulations [1,2,3]. Different crystal structures can affect the physicochemical properties of APIs, such as melting point, dissolution rate, and solubility, thereby influencing bioavailability, stability, and other performance characteristics. The solid forms include polymorphs and solvatomorphs. Solvatomorphs are crystal forms generated by the incorporation of APIs with one or more solvent molecules in the crystal lattice; defined as pseudopolymorphs and crystalline solvates, they involve the same chemical entity and incorporate solvent molecules into the crystal lattice, resulting in a different structure. The formation of a solvatomorph is important during the API development process [4,5]. Therefore, investigating and developing solid forms is crucial for the API. Furthermore, the rational development and application of solid forms can guide the design of manufacturing processes to ensure the safety and efficacy of the medication [6,7].

**Figure 1:**
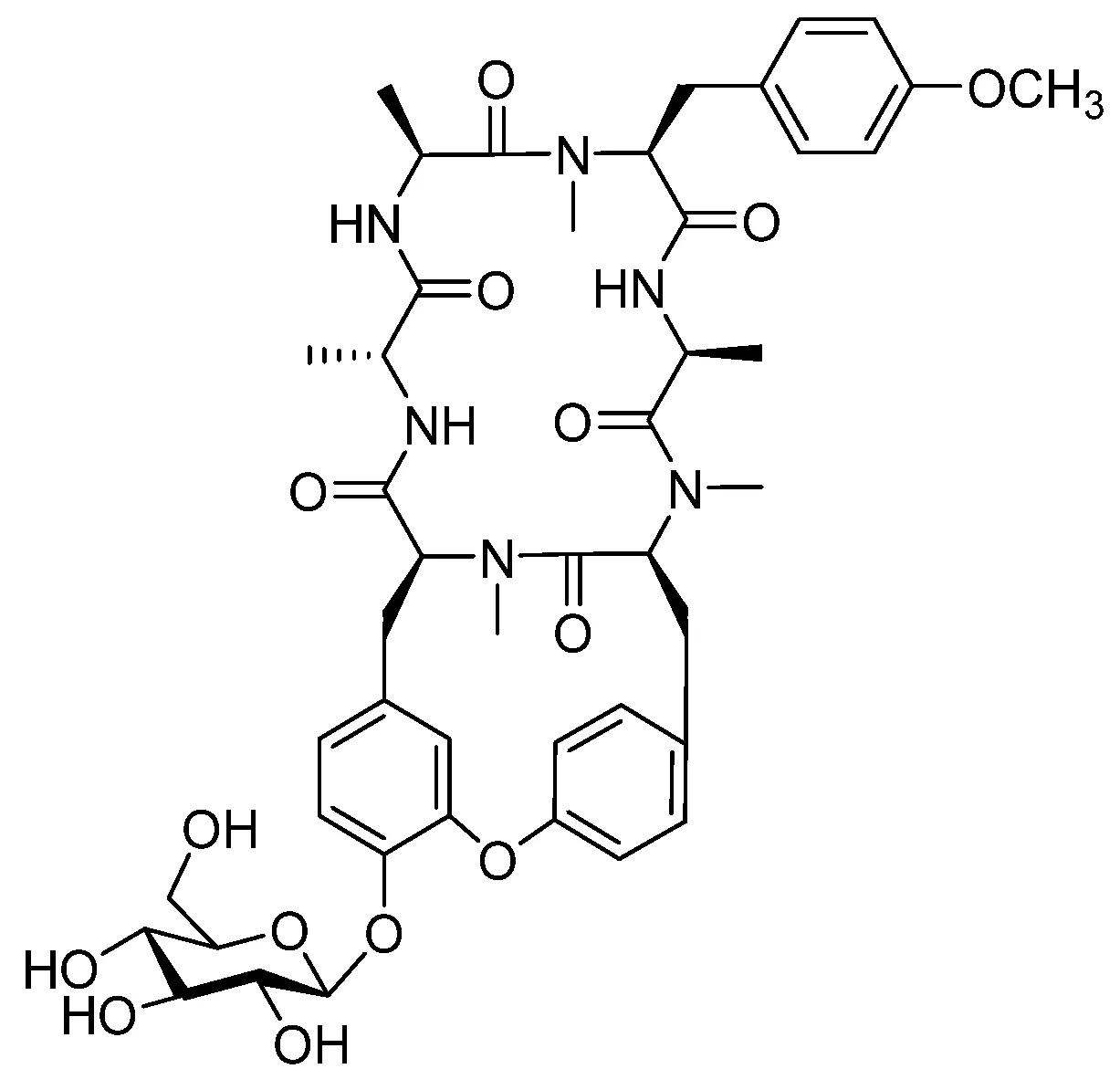
Chemical structure of RA-XII.

RA-XII (C_46_H_58_N_6_O_14_, CAS Registry No. 143343-98-0, Figure 1) is a potential anti-tumor candidate that is being researched and developed for the treatment of colorectal cancer in our lab. Our previous studies have shown that RA-XII could suppress protective autophagy by regulating mTOR and NF-κB pathways in colorectal cancer cells, restore colitis damage through regulating gut microbiome to exert anti-inflammatory effects against colorectal cancer, and inhibit colorectal neoplasm growth and metastasis by reducing SREBP 1 expression, thereby inhibiting de novo fatty acid synthesis [8,9,10]. To improve water solubility and permeability, RA-XII was developed into an injectable formulation and oral solid dispersion, respectively [11,12]. However, there were no reports on the solid form of RA-XII, and investigations into its crystal forms remain blank. Therefore, an in-depth study of the solvatomorphic diversity and formation mechanisms of RA-XII as an API was warranted.

In this work, six forms of RA-XII were prepared via various crystallization methods, in which single crystals of three forms (Forms I, II and V) were obtained for the first time. Stability evaluation of RA-XII solvatomorphs and its amorphous form under different conditions was carried out, and their thermodynamic stability was established through differential scanning calorimetry (DSC) and thermogravimetric analysis (TGA). Additionally, the intermolecular interaction, molecular conformation, and packing arrangement of three single crystals (Forms I, II and V) were analyzed through structural analysis and energy calculations. It is worth mentioning that we took advantage of molecular electrostatic potential (MEP) analyses and dynamics simulations to elucidate the interactions between RA-XII and two solvent molecules. Finally, the formation mechanism of RA-XII solvatomorphs was discussed. These results provide valuable references for the API solvatomorph control of RA-XII.

## 2. Materials and Methods

### 2.1. Materials

RA-XII (API, purity > 98.5%) was isolated and prepared by our Pre-lab team. All organic solvents (analytical purity grade) were purchased from Shanghai Titan Scientific Co., Ltd. (Shanghai, China). Distilled water was prepared by Mili-Q in the laboratory.

### 2.2. Preparation of RA-XII Solvatomorphs and Amorphous Form

RA-XII was dissolved in 30% ethanol-water solution at room temperature and stirred for 4 h, then subjected to evaporation crystallization at 55 °C until an adequate quantity of crystals had precipitated by reducing pressure. Subsequently, the precipitated solid was collected by vacuum filtration, drying at 55 °C, and Form I was obtained. Form V was produced via slurry crystallization in appropriate amounts of ethyl acetate at 40 °C for 12 h. Then, the solids were dried at 25 °C in an airing cupboard. Form VIII was prepared by dissolving RA-XII in 50% ethanol-water at 50 °C and gradually cooling to 20 °C at a controlled rate of 0.5 °C/min until an adequate quantity of crystals had precipitated, and the rest solvent was evaporated at 150 °C and −0.8 bar in a vacuum oven. Detailed preparation methods for other forms are summarized in Table S1.

### 2.3. Powder X-Ray Diffraction

Powder X-ray diffraction (PXRD) was performed to identify the solid crystalline samples. Data were acquired using a SmartLab SE (Rigaku Corporation, Tokyo, Japan, generator: voltage of 40 kV) from 2° to 40° at a scan rate of 10°/min with CuKα radiation (1.54060 Å). The data were analyzed by using Materials studio 2020 software. Variable temperature PXRD (VT-PXRD) was performed on a Bruker D8 advance diffractometer (Bruker AXS K.K., Kanagawa, Japan) at each temperature point, heating at a rate of 10 °C/min from 25 °C to 200 °C and holding for 10 min at each point. Other conditions of VT-PXRD are the same as those of PXRD.

### 2.4. Spectroscopy Analysis

Fourier-transform infrared spectroscopy (FT-IR) was performed using a Bruker alpha II Spectrum (Tensor 27) with 20 scans per spectrum. The spectra were determined over the wavelength of 4000−400 cm^−1^.

Raman spectroscopy (RM) was performed using a React Raman HR evolution spectrometer (LabRAM HR evolution, Horiba scientific, Longjumeau, France) at an excitation wavelength of 532 nm with laser power consumption at 200 nw. The spectra were recorded from 4000 to 50 cm^−1^ at a resolution of 2–3 cm^−1^. Data were analyzed using Origin 2021.

### 2.5. Thermal Analysis

DSC was performed using a Netzsch 3500 Sirius instrument (NETZSCH, Selb, Germany), data acquisition and analysis were performed using Proteus software (Version 9.7), and 5 mg samples were analyzed at a heating rate of 10 °C/min under a nitrogen flow of 50 mL/min. The temperature range was 25–500 °C.

TGA was conducted using a TGA 4000 system (PerkinElmer, Waltham, WA, USA), data acquisition and analysis were performed using Pyris software (Version 11). RA-XII samples (5 mg) were heated at a rate of 10 °C/min from 30 to 800 °C under a nitrogen atmosphere.

### 2.6. Morphology Analysis

The crystal morphology of RA-XII was characterized by using an optical microscope (Eclipse Ei R, Nikon) equipped with a BE2 PLAN camera (Nikon CORPORATION, Tokyo, Japan). Additional analyses were performed by using a scanning electron microscope (SEM) (Regulus-8100, Hitachi High-Tech Corporation, Tokyo, Japan).

### 2.7. Stability Experiments

Stability experiments were performed by storing the solvatomorphs and an amorphous form in controlled environments at 40 °C and 60 °C, under strong glare (4500 ± 500 lx) and 90% RH at 25 °C for 30 days, respectively. The pre-experimental stability was assessed under four conditions for 10 days. The collected samples were characterized by PXRD.

### 2.8. Single-Crystal Growth and Determination

Single crystals of Form I obtained in 2.2 were prepared through the evaporation of a 30% ethanol-water solution at 40 °C for 3 days. Single crystals of Form II obtained in 2.2 were prepared by the evaporation of ethyl acetate-water (1:2) solution in a ventilation hood at room temperature (rt). Single crystals of Form V obtained in 2.2 were prepared via the antisolvent method, i.e., an appropriate amount Form V was dissolved in methanol and crystallized by diffusion of ethyl acetate by vaporing into the saturated methanol solution at rt. Single-crystal X-ray diffraction was conducted on a XtaLAB Synergy-DW single-crystal X-ray diffractometer (Rigaku Corporation, Tokyo, Japan) using Cu Kα radiation (λ = 1.54178 Å). The structures were subsequently solved using the ShelXT structure solution program within the Olex2 platform.

### 2.9. Dynamics of Crystallization

This study determined the solubility of API forms in water at different temperatures. The Van’t Hoff equation was used to calculate the actual solubility of the solution, taking into account the intermolecular interaction between the water and RA-XII molecules, and substituting the enthalpy of dissolution (standard molar enthalpy of dissolution, Δ*Hd*) and the entropy of dissolution (standard molar entropy, Δ*Sd*), resulting in the following Van’t Hoff equation:

$$\ln x=\;-\frac{\Delta \mathrm{Hd}}{\mathrm{RT}}+\frac{\Delta \mathrm{Sd}}{R}$$

In the above equation, the heat of dissolution is the heat released or absorbed when 1 mol of solute dissolves in a solvent under specific temperature and pressure conditions. Under isobaric conditions, the heat of dissolution is equal to the change in enthalpy and may also be referred to as the enthalpy of dissolution (Δ*Hd*). R represents the universal gas constant (8.3145 J·K^−1^·mol^−1^), and T represents the corresponding absolute temperature (K). Plot ln x against 1/T to calculate the enthalpy of dissolution (Δ*Hd*, kJ·mol^−1^) and the entropy of dissolution (Δ*Sd*, kJ·mol^−1^·K^−1^). The Van’t Hoff analysis was applied to obtain Δ*Hd* and Δ*Sd* for each solvatomorph. This model assumes ideal-solution behavior and invariant solid phase during the experiment; conditions are not fully satisfied for these solvatomorphs, which may undergo partial desolvation in aqueous media.

The experimental procedure is as follows: Excess samples are added to 25 mL of water at different temperatures, with water bath temperatures of 293.15 K, 303.15 K, 313.15 K, 323.15 K, and 333.15 K, respectively, and a stirring speed of 75 rpm/min. After stirring for 48 h, a 2 mL aliquot was withdrawn and centrifuged at 13,000 rpm for 15 min; the content of RA-XII was determined by HPLC. All experiments were performed in triplicate. Values are expressed as mean ± standard deviation.

### 2.10. Equilibrium Solubility Study

The solubility of RA-XII solvatomorphs and an amorphous form was determined using the shake-flask method in distilled water (pH 7.0) and different pH buffer solutions (pH: 1.2, 4.5, 5.5, 6.8, 7.4) at 37 °C. Excess samples were added to 25 mL of those solutions in a centrifuge tube, preheated at 37 °C, and the resulting slurry was subjected to a water bath shaker. At a predetermined time point (48 h), a 2 mL aliquot of the slurry was withdrawn. The suspensions were centrifuged at 13,000 rpm for 15 min and measured for content by HPLC. The experiment was repeated three times in parallel, and values were presented as mean ± standard deviation.

### 2.11. Intrinsic Dissolution and pH Study

An intrinsic dissolution study was carried out for 24 h in distilled water and in PBS buffer solution (pH 7.4) on a *Chinese Pharmacopeia* certified Dissolution Testing Apparatus FADT-1200RC (FOCS, Shanghai, China) using a paddle method. Standard curves for six forms of RA-XII in two solutions were obtained by HPLC at 225 nm. A 30 mg aliquot of the compound was added to the dissolution vessels, and the paddles were lowered into the dissolution vessel containing 500 mL of distilled water at 37 °C and stirred at 75 rpm. At set point time intervals, an aliquot (2 mL) was withdrawn, and an equal volume of solution added. After centrifuging at 13,000 rpm for 15 min, the concentration was determined by HPLC. pH values of the solutions were measured before the addition of the samples and after 1 h using an Inesa PHS-3C+ pH meter (Inesa Scientific Instrument Co., Ltd., Shanghai, China). The experiments were repeated in triplicate with two solutions.

### 2.12. High-Performance Liquid Chromatography (HPLC)

HPLC was performed using an Agilent LC1260 system and a ZORBAX Extend C18 5 μm column (4.6 mm × 250 mm) (Agilent Technologies, Inc., Santa Clara, USA) for solubility and dissolution study. The mobile phase consisted of methanol (B) and distilled water (A). The experiment was carried out on a gradient elution program: 0–2 min, 48% B; 2–15 min, 48−52% B; 15–20 min, 52−70% B; 20–25 min, 70−95% B; 25–26 min, 95−48% B; and 26–30 min, 48% B at a wavelength of 225 nm. The injection volume was 10 μL, and the flow rate was 1.0 mL/min.

### 2.13. Molecular Simulation

Hirshfeld surface analysis and two-dimensional fingerprint plots were performed to investigate intermolecular interactions within the crystal structure using Crystal Explorer 17.5 [13]. Additionally, the lattice energies of the three solvatomorphs were calculated using the same software. Molecular geometries were optimized using Chemdraw 3D (Version 16.0) based on Gaussian 09 using the DFT=B3LYP method on the set of 6-311G+ (d, p) level of theory [14,15]. The molecular electrostatic potential (MEP) results were computed by using Multiwfn 3.8 and visualized with VMD 1.9.3 software [16,17].

Molecular dynamics (MD) simulations were performed using the Forcite module in COMPASS II force field within Materials Studio 20 (Accelrys Software Inc., USA), according to reference [7]. The COMPASS II force field extends the coverage of functional group parameters for a wide variety of drug molecules; these parameters have been validated by quantum chemical calculations and condensed-phase (crystal, liquid) experimental data. It is well-suited for simulating the crystal and solvated states (including lattice energy, unit cell optimization, and molecular dynamics) of organic molecules and liquid-phase simulations of cyclopeptide molecules of medium molecular weight. Therefore, the COMPASS II was selected for the present modeling of RA-XII (*m*/*z* 918) due to its excellent applicability to molecules of this molecular weight range [18]. A series of amorphous cells were constructed using RA-XII and solvent molecules, which were initially optimized to minimize energy, based on the solubility. These amorphous cells represented ethanol-water (50:50, *v*/*v*) and ethyl acetate solutions, respectively. The rationales behind the RA-XII: solvent ratios were both based on a solution ratio of 100:1, which was used in the preparation of solvates. After geometric optimization, MD simulations were conducted using an NVT ensemble at 298.15 K, with a simulation duration of 500 ps and a time step of 1 fs. Temperature regulation was achieved via a Nosé−Hoover−Langevin (NHL) thermostat. Subsequently, 10 ns of MD simulation was conducted using an NPT ensemble at 298.15 K. Radial distribution function (RDF) results were subsequently analyzed using the Forcite module.

Solvation free energy was calculated using the Forcite module with the COMPASS II force field, which was the sum of three components: the ideal free energy (ΔGid) in a vacuum with charges removed, the van der Waals free energy (ΔGvdw) with the addition of a neutral molecule to the solvent, and the electrostatic free energy (ΔGelec) upon adding charges to the solute [7]. Periodic-cell calculations yielded solvation free-energy values for the amorphous cells. All raw outputs were normalized on a per API molecule basis by dividing the unit-cell total solvation free energy by *Z*, where *Z* corresponds to the number of RA-XII molecules.

## 3. Results and Discussion

### 3.1. Characterization

#### 3.1.1. Powder X-Ray Diffraction (PXRD)

**Figure 2:**
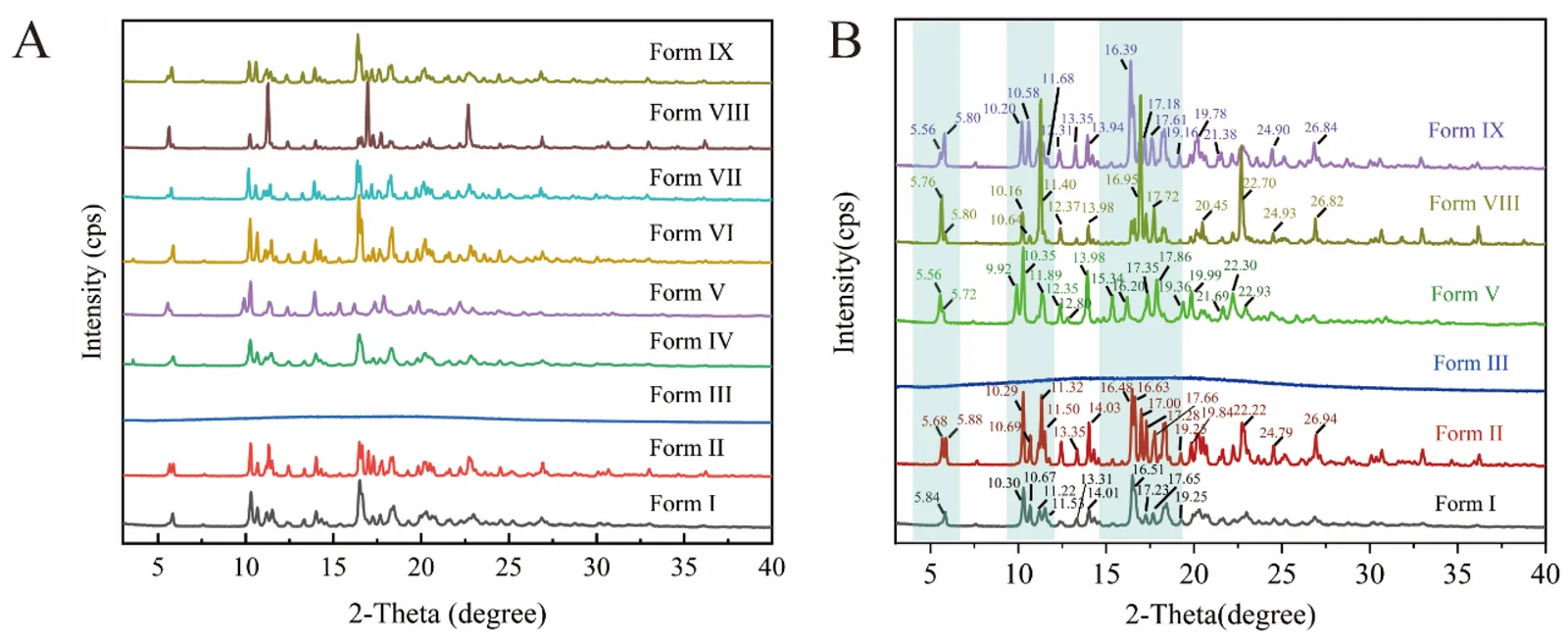
Characteristic PXRD patterns of RA-XII solvatomorphs and amorphous form, (**A**) nine forms, (**B**) enlargement for six forms.

In this study, we successfully screened nine distinct forms of RA-XII (Table S1). Firstly, the nine forms (Forms I–IX) were characterized using PXRD, as shown in Figure 2A and Table S2. An in-depth study of six forms (Forms I–III, V, VIII and IX) was carried out according to the pre-experimental stability because the purity of RA-XII in Forms IV, VI and VII decreased after 10 days of storage under four conditions (Figure S1, Table S3), indicating that Forms IV, VI and VII were unstable. The FT-IR, morphology characterizations and thermal analysis of Forms IV, VI and VII are shown in Figures S2–S4. Except for Form III, which showed no diffraction peaks, the other five forms (Forms I, II, V, VIII and IX) demonstrated clearly distinct characteristic peaks, particularly in the green-cyan areas of Figure 2B. For Form I, the main diffraction peaks appear at 5.84°, 11.22° and 16.51°; for Form II, the peaks are at 5.68°, 5.88°, 11.32°, 17.00 and 19.84°; for Form V, the peaks are at 5.56°, 9.92°, 11.89°, 12.80° and 17.35°; for Form VIII, the peaks are at 5.76°, 10.16°, 11.40° and 17.72°; and for Form IX, the peaks are at 5.80°, 10.58°, 11.68°, 17.18° and 19.16°. However, Forms I and II have common peaks at about 10.30°, 17.20°, 17.65°and 19.25°. Moreover, they share similar peaks with Forms VIII and IX at approximately 12.30°, 13.95°, 24.90° and 26.80°. Most peaks of Form V differ evidently from those of Forms I, II, VIII and IX. Form III displays no diffraction peaks in the range of 3~40°, which suggests that Form III is an amorphous state. Therefore, all the characteristic peaks indicate the discovery of six new forms for RA-XII for the first time. Because the PXRD patterns of the six forms showed little variation, we presumed that their structures might be those of channel-type solvates, suggesting that the main structural differences between the various forms depend on the types and proportions of solvents within the channels.

#### 3.1.2. Thermal Analysis

**Figure 3:**
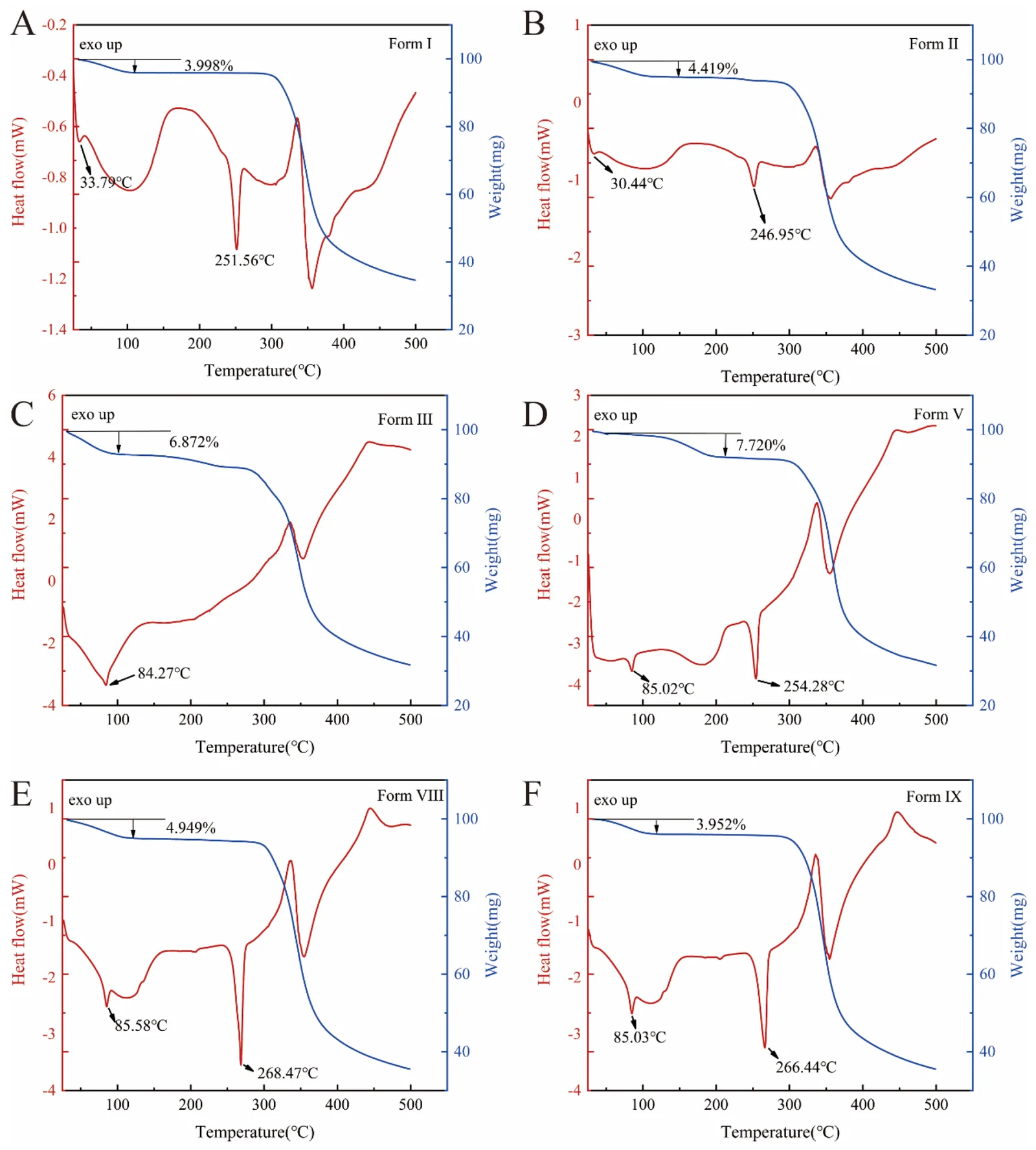
Characterization of RA-XII solvatomorphs and amorphous form. TGA (blue lines) and DSC (red lines) analyses for (**A**) Form I, (**B**) Form II, (**C**) Form III, (**D**) Form V, (**E**) Form VIII, and (**F**) Form IX.

Then, to compare the thermodynamic properties of the five solvatomorphs and the amorphous form, DSC and TGA analyses were performed. As illustrated in Figure 3 and Table 1, their TGA curves revealed double-weight loss stages, with one weight loss stage beginning at approximately 25 °C and ending at 110 °C and another weight loss stage beginning at approximately 310 °C. The percentages of weight loss were 3.998% for Form I, 4.419% for Form II, 6.872% for Form III, 7.720% for Form V, 4.949% for Form VIII, and 3.952% for Form IX, respectively, indicating that all forms are solvatomorphs, apart from Form III. According to the molecular weights of RA-XII and the solvent molecule, the weight loss of trihydrate was 3.7–5.5%, while the weight loss of mono-EtOAc-solvate was 7.7–8.7%. The results showed Forms I, II, VIII and IX were tri-hydrates and Form III was amorphous with solvent, while Form V was mono-EtOAc-solvate by calculating stoichiometric ratios. DSC results suggested that five samples differ in melting points: 251.56 °C for Form I, 246.95 °C for Form II, 254.28 °C for Form V, 268.47 °C for Form VIII, and 266.44 °C for Form IX, while Form III had no fixed melting point, indicating that Form III was amorphous. Combined with the TGA results, except for Form III (amorphous), the other five forms transformed into dehydrates following solvent loss in the first stage, in which the melting points in the DSC analysis should be the melting points of Forms I, II, V, VIII and IX dehydrates. Furthermore, among these dehydrates of solvatomorphs (Forms I, II, V, VIII, IX) and the amorphous form (Form III), based on the heat-of-fusion rule [19,20] and △H_fus_, the dehydrate of Form VIII was supposed to be the most thermodynamically stable, followed by dehydrates of Form IX and Form II in descending order following △H_fus_. The melting enthalpy-based stability ranking was established using thermal data of the dehydrated of solvatomorphs and amorphous form.

#### 3.1.3. Morphology Analysis

**Figure 4:**
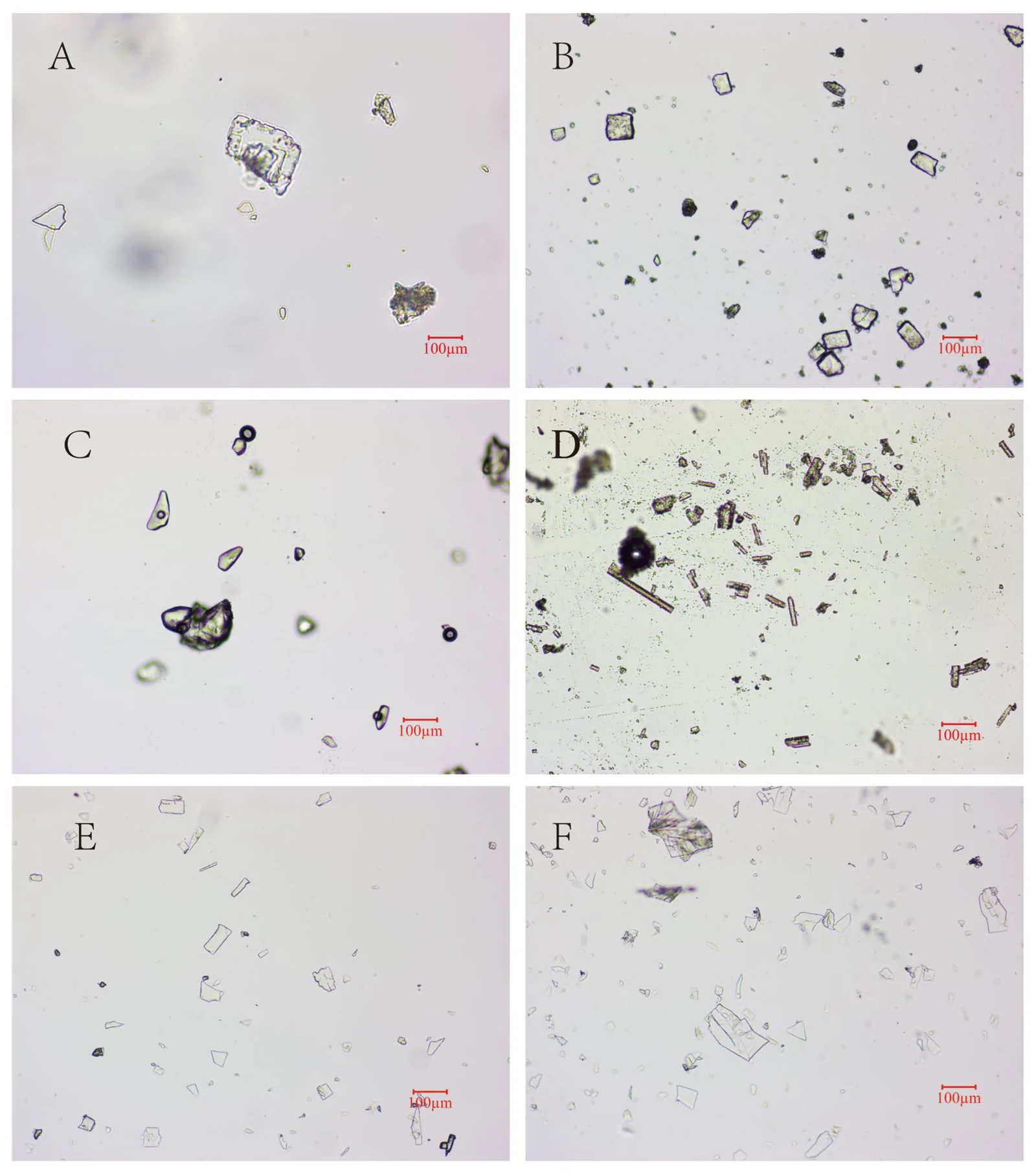
Microscopy images of (**A**) Form I, (**B**) Form II, (**C**) Form III, (**D**) Form V, (**E**) Form VIII, and (**F**) Form IX.

**Figure 5:**
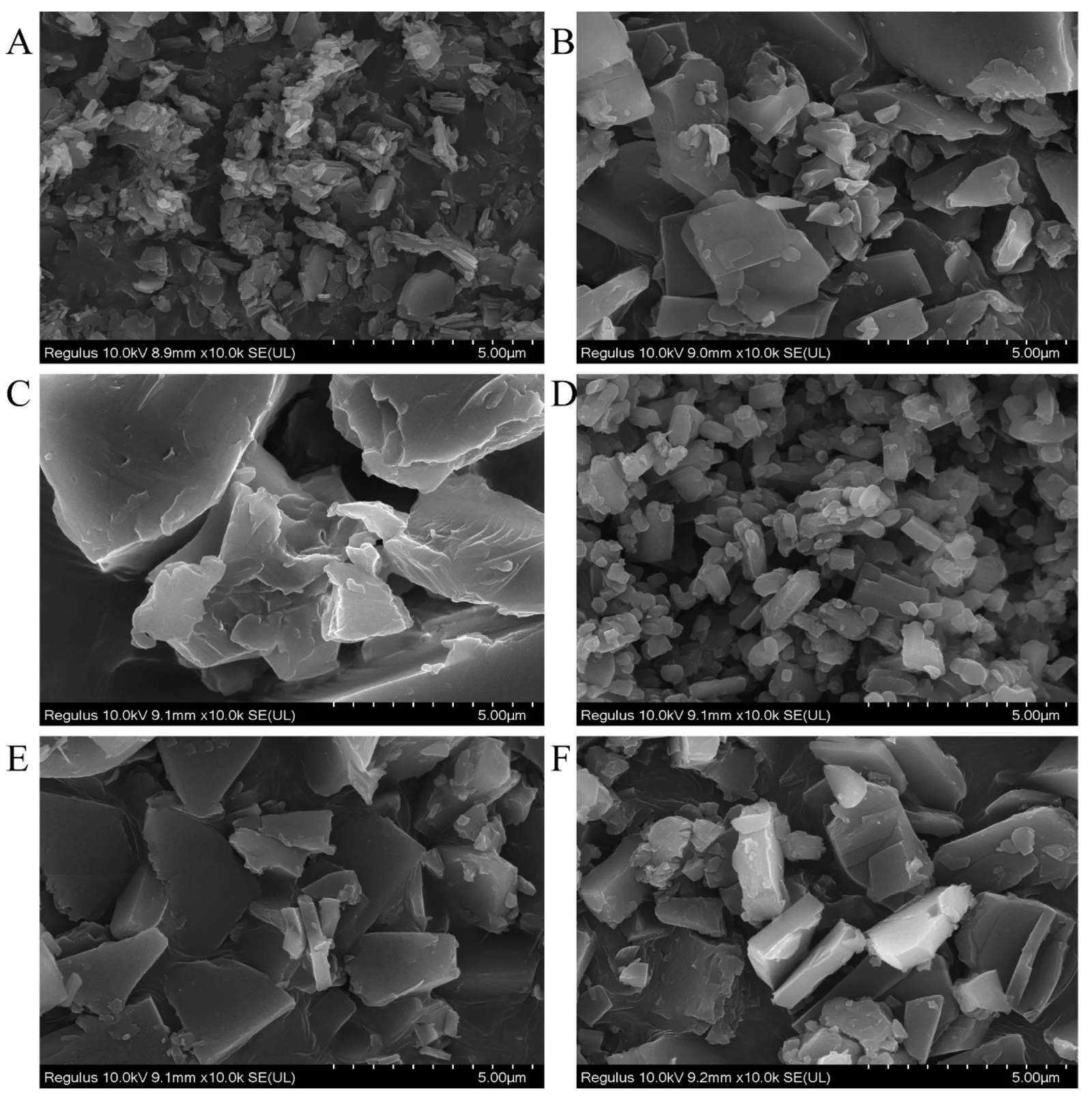
SEM images of (**A**) Form I, (**B**) Form II, (**C**) Form III, (**D**) Form V, (**E**) Form VIII, and (**F**) Form IX.

Additionally, the morphological differences and SEM among the five solvatomorphs and amorphous form were analyzed, as shown in Figure 4 and Figure 5. Microscopy images of 100 µm in size showed that Form I had a square plate-like structure, Form II a square blocklike structure, Form III an irregular structure, and Form V a rod-shaped structures, while Forms VIII and IX both had square flakelike structures. SEM images showed subtle differences, i.e., Forms II, VIII and IX as flakelike morphologies in size of 5 µm, Forms I and V as fasciculate-like morphologies, and Form III as irregularly stony-like morphologies.

#### 3.1.4. Spectroscopy Analysis

**Figure 6:**
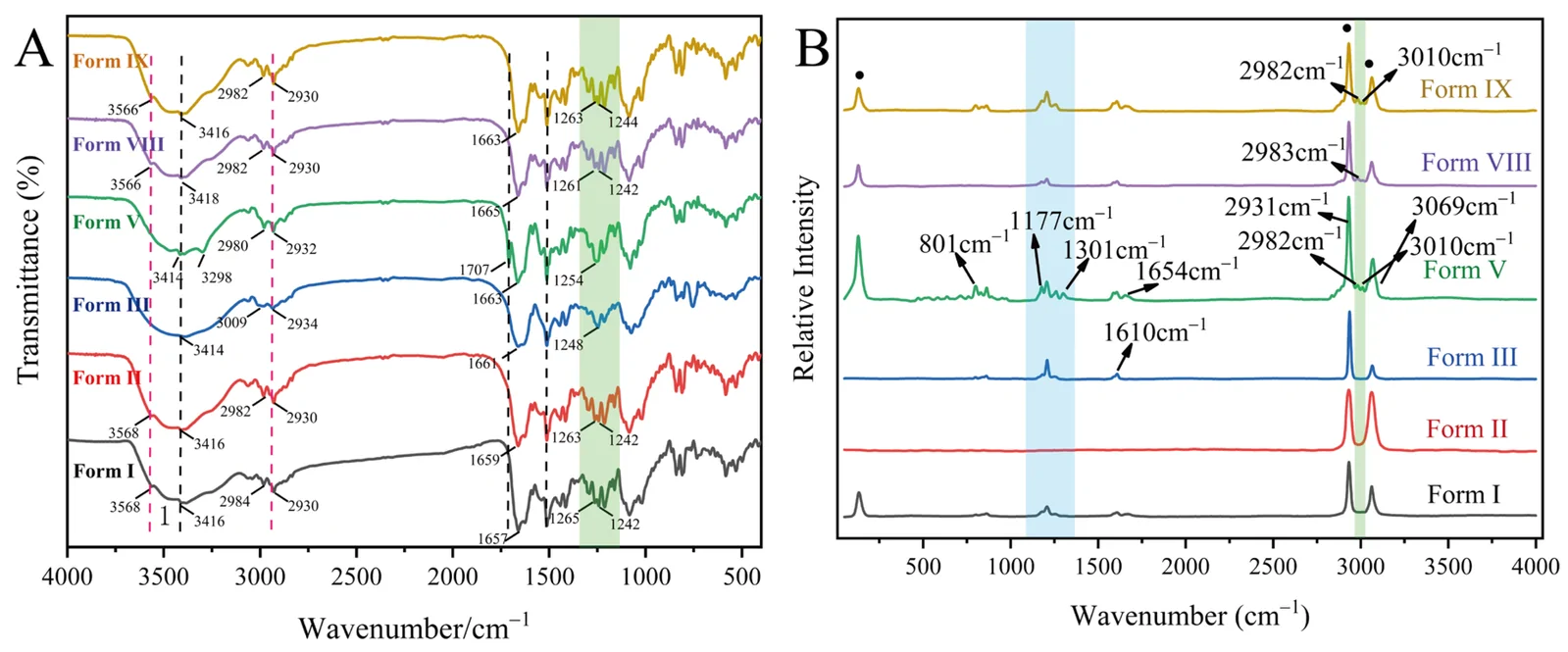
(**A**) FT-IR spectra of six forms. (**B**) Raman spectra of six forms.

To more meticulously characterize and compare the five solvatomorphs and an amorphous form in terms of chemical structure, spectral analysis was performed. The infrared spectra (FT-IR) details of the six forms with the shifted peaks are highlighted in Figure 6A, and the summary as illustrated in Table 2. The C=O stretching vibrations were demonstrated at 1657 cm^−1^ for Form I, shifting to 1659, 1661, 1663, 1665 and 1663 cm^−1^ for Forms II, III, V, VIII and IX, respectively. However, a sharp singlet peak appeared at 1707 cm^−1^, and a broad singlet peak appeared at 3298 cm^−1^, suggesting a relationship with ethyl acetate, the crystallization solvent for Form V. For Forms I, II, VIII and IX, a strong small singlet peak appeared at 3566–3568 cm^−1^, corresponding to the O–H stretching vibration. Additionally, the C–H out-of-plane bending vibration shifts ranged from 2930 cm^−1^ to 3009 cm^−1^ in six forms, while there was not much distinction in the chemical shifts. The N–H out-of-plane bending vibration peak at 3059 cm^−1^ in Form III was split into two peaks in the other five Forms. Likewise, for Forms I, II, VIII and IX, there were double peaks at 1265 cm^−1^ and 1242 cm^−1^, 1263 cm^−1^ and 1242 cm^−1^, 1261 cm^−1^ and 1242 cm^−1^, and 1263 cm^−1^ and 1244 cm^−1^, respectively, corresponding to the C–O stretching vibration, while only one peak appeared at 1248 and 1254 cm^−1^ in Forms III and V, respectively. Most peaks of Form I differed inconspicuously from those of Forms II, VIII and IX, indicating their FT-IR spectra are similar and that they share comparable crystal structures and intermolecular interactions, consistent with the findings of the PXRD analysis mentioned above.

Furthermore, RM was conducted to thoroughly characterize the five solvatomorphs and an amorphous form. The results are presented in Figure 6B, and the summary is illustrated in Table 3. RM showed significant differences among these RA-XII solvatomorphs and the amorphous form. Form I exhibited three characteristic peaks at 3061.85 cm^−1^, 2931.83 cm^−1^, and 133.52 cm^−1^, while the RM for Form II was similar at 3061.85 cm^−1^ and 2931.83 cm^−1^, at 3061.85 cm^−1^ and 2931.83 cm^−1^ for Form VIII, and at 3063.05 cm^−1^ and 2931.83 cm^−1^ for Form IX, respectively, in which these peaks indicated the similarity of their crystal structures. Form V expressed four characteristic peaks at 1654.00 cm^−1^, 1301.00 cm^−1^, 1177.32 cm^−1^, and 800.59 cm^−1^, while Forms II and VIII were missing these characteristic peaks, but slight variations were observed for Forms I, III and IX. However, for Form II, only the strong double peak appeared at 2931.83 cm^−1^ and 3061.85 cm^−1^, comparing with Forms I and V. There were one or two smaller vibration peaks between 3069.04 cm^−1^ and 2930.60 cm^−1^ for Forms V, VIII and IX, while Forms I, II and III lacked these peaks (the green areas in Figure 6B).

### 3.2. Solubility and Intrinsic Dissolution Studies

Firstly, we measured solubility of the five solvatomorphs and an amorphous form in water by the dynamic method. The solubility data are graphically presented in Figure S5. The concentration was determined by HPLC with the calibration curve method, which was linear over the range of 0.0012–0.3136 mg/mL (R^2^ = 0.9998). The limit of detection (LOD) and limit of quantification (LOQ) values were 0.2 and 0.4 μg/mL, respectively. Solubility of the five solvatomorphs and an amorphous form followed a positive correlated increase with temperature (Figure S5). Forms VIII and IX exhibited higher solubility by ranking as follows: Form IX > Form VIII > Form V > Form II > Form I > Form III. Using the Van’t Hoff equation to calculate the Δ*Hd* and Δ*Sd* [21], for Form IX, both Δ*Hd* and Δ*Sd* were found to be greater than those of the other forms. For Form I and Form III (amorphous), Δ*Hd* was greater than that of Form II and Form V. The Δ*Sd* and Δ*Hd* values for all forms were positive, indicating that their dissolution process in water is endothermic; heating therefore promotes RA-XII dissolution (Figure S6 and Table S4).

Then, apparent solubility and dissolution kinetics experiments in water and different pH buffer solutions were performed to further explore stability and solubility. In apparent solubility experiments, all samples of five solvatomorphs and an amorphous form were dissolved at 37 °C in distilled water with a shaking rate of 75 rpm for 48 h. The results are shown in Figure S7 and Table S5. Form II exhibited the highest solubility of 141.59 µg/mL. The solubility in descending order was as follows: Form II > Form VIII > Form I > Form IX> Form III > Form V. Forms I, II, VIII and IX all exhibited high solubility. It is presumed that this phenomenon was related to the presence of hydrogen bond networks between water and RA-XII molecules within these forms, with enhanced hydrogen bonding interactions between RA-XII and water molecules. By contrast, Form V showed the lowest solubility, which was presumed to be attributed to the hydrophobic ethyl acetate molecules inside the form, which constructed a hydrophobic layer with RA-XII molecules. Form III shows the lower solubility in water. It is presumed that the amorphous form lacked a regular arrangement, which could be regarded as thermodynamically super-cooled and exhibiting miscible gaps with water. When excess amorphous form dissolves in water, it can cause a supersaturation phenomenon, and the equilibrium concentration is achieved rapidly, showing liquid–liquid phase separation (LLPS) and forming a two-phase system with lower free energy: a drug-rich phase and a continuous supersaturated aqueous phase [22,23]. Nevertheless, the solubility of the amorphous form (Form III) was abnormally lower than that of other forms and presumed to originate from aggregation and gelation effects [24]. Residual solvent and hydrophobic interactions facilitate the formation of surface gel layers or large particles, blocking interactions between RA-XII and water molecules. LLPS serves as a key mechanism for maintaining high supersaturation.

For the dissolution kinetics experiments, we measured the solubility at predetermined time points over a 24 h period and expressed the rate of dissolution in terms of the fitting equations within 24 h. The results are shown in Figures S8 and S9 and Table S6. Form IX exhibited the fastest dissolution rate in distilled water to flat solubility. However, Form II displaying the maximum dissolution among the five solvatomorphs and amorphous form, and Form I exhibited the lowest dissolution rate. The slower dissolution rate of Form V was presumed to be related to its transformation into Form I (Figure S8). Similarly, Form I exhibited the fastest dissolution rate in pH 7.4 PBS buffer to flat solubility. Form VIII displayed the maximum dissolution (Figure S9). These results demonstrated that Form II and Form VIII exhibited better solubility and had advantages of six forms in dissolution. The amorphous form showed distinct dissolution behaviors in the two media. The amorphous (Form III) was in a supersaturated state in water, exhibited LLPS, and reached equilibrium concentration relatively quickly (Figure S8). However, in pH 7.4 PBS, phosphate ions induce salting-out, disrupting the two-phase system. This dissolution concentration exceeded the LLPS threshold, leading to a prolonged time to attain equilibrium concentration (Figure S9). Therefore, the dissolution behaviors are consistent with those reported [24].

**Figure 7:**
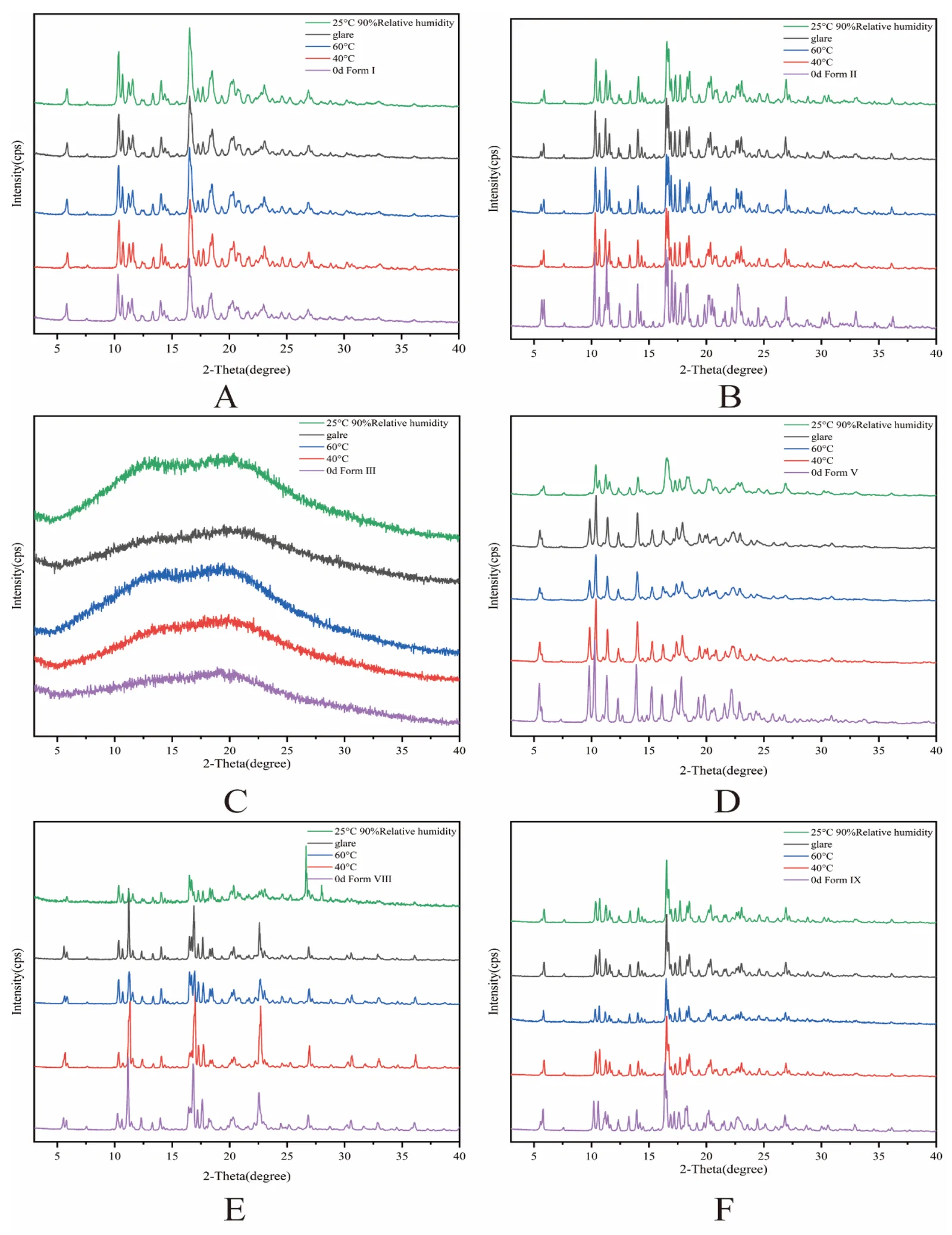
PXRD results of stability experiments with five RA-XII solvatomorphs and an amorphous form for 30 days. (**A**) Form I, (**B**) Form II, (**C**) Form III, (**D**) Form V, (**E**) Form VIII, (**F**) Form IX.

Afterwards, accelerated stability experiments were conducted under the conditions of 40 °C, 60 °C, 4500 lx glare, and 90% RH for 30 days to further evaluate the stability of the five solvatomorphs and amorphous form, respectively. The results as presented in Figure 7. Except for Form II, the diffraction peaks of four RA-XII solvatomorphs and the amorphous form (Forms I, III, V, VIII and IX) remained unchanged at conditions of 40 °C, 60 °C, and 4500lx glare for the 30-day period, indicating that the five forms were stable under these conditions (Figure 7A−F). However, under 90% RH conditions (high humidity at room temperature), the characteristic peaks of Form I appeared in those of Forms V and VIII, indicating that Forms V and VIII were completely transformed into Form I within 30 days, but Forms II and IX were partially transformed into Form I. This result suggested that Form I was more stable than Forms II, V, VIII and IX in high humidity. However, Forms I and III had were not converted to any other forms within 30 days under four conditions, and the amorphous sample (Form III) was not converted into a crystalline form. These results demonstrated that Forms I and III exhibited superior stability under conditions of high temperature and humidity. The apparent solubility in different pH buffer solutions is shown in Figure S10. Form II showed the lowest solubility at pH 5.5, but the highest solubility in water, exhibiting an initial increase followed by a decrease. Forms III, V, and IX exhibited overall decreasing trends, particularly from pH 4.5 to pH 7.4. The solubility of Forms I and VIII showed irregular differences among these buffers. In the above experiments, all results indicated that Form I was the most stable form. Although Forms II and VIII demonstrated equilibrium solubility comparable to or higher than that of Form I, they transformed into Form I under 90% RH conditions (high humidity at room temperature) in our stability experiment (Figure 7B,E). This was not conducive to storage under high humidity at room temperature.

Lastly, the hygroscopicity analysis of the six forms was conducted under the conditions of 90% RH at 25 °C for 30 days to investigate potential moisture absorption, with the results displayed in Figure S11. The weight of Forms III and V increased significantly at 90% RH in 5 days, whereas Forms I, II, VIII and IX exhibited insignificant moisture absorption with below 0.75% weight increase. At 30 days, the adsorption capacity of all forms was below 1.0%. It was speculated that this was due to a form transition, desorption, or water loss occurring when the sample absorbed moisture to a certain extent (<0.75%), resulting in a decrease in weight change. These results demonstrated that, except for Form III, Form V exhibited higher moisture absorption than Form I, which might be related to Form V transforming into Form I under the high-humidity conditions of 90% mentioned above. Form V is a mono-EtOAc-solvate (Section 3.1.2), In the early stages of exposure to a high-humidity environment (90% RH), no hydrophilic adsorption sites were exposed on the surface of Form V; However, the moisture-induced weight gain rate at the first 5 days was significantly higher than that of Form I, which already contained water channels. As the exposure time increased, water molecules from the environment gradually diffused into the lattice channels of Form V, slowly replacing the ethyl acetate guest molecules within the channels, ultimately transforming it completely into Form I. This form transition was a slow isomorphous transformation that does not result in macroscopic crystal collapse. At 30 days, the total weight increase in the fully transformed sample was almost exactly the same as the final moisture-induced weight gain of Form I under identical conditions. Therefore, this phenomenon might be attributed to water molecules replacing the ethyl acetate molecules in Form V under high-humidity conditions, and the weight increases of Form V and Form I were consistent after 30 days.

**Figure 8:**
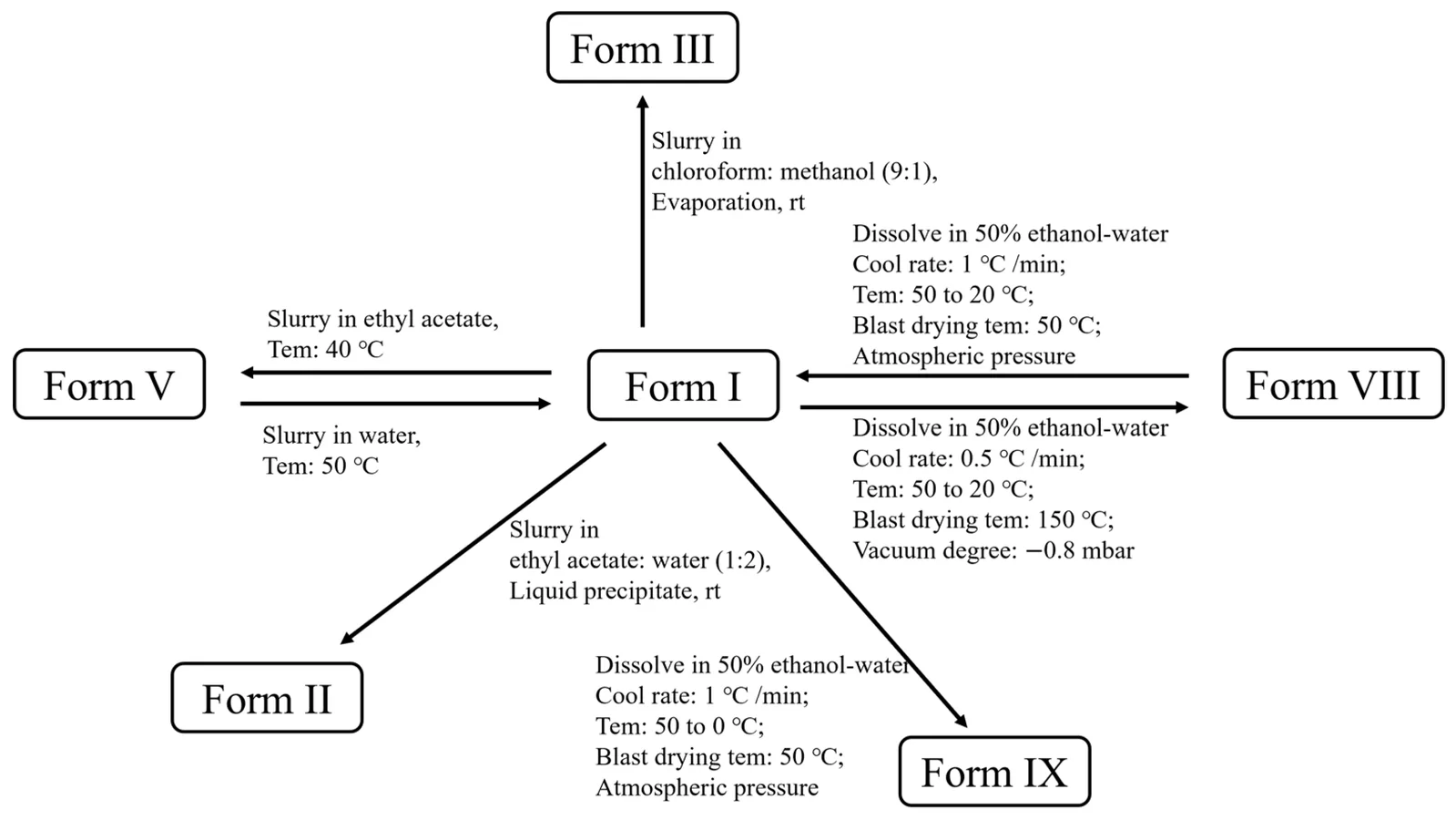
Transformations among six RA-XII forms.

Numerous water activity experiments in various organic/water solvent systems and under different conditions, including cooling rate, vacuum degree, and temperature, were then designed to understand the complex transformation relationship of these forms [25], as summarized in Figure 8. The relevant experimental conditions are summarized in Table S1. The results indicated that Form I appeared to be the predominant species in water, and the transition of Forms V and VIII into Form I was confirmed with the conclusion of the stability experiment under the high-humidity conditions mentioned above. Furthermore, an experiment with a gram-scale API of Form I was performed, consistently demonstrating stability and reproducibility (Figure S12). The water activity threshold was determined to be approximately 0.30–0.95. This API Form I satisfied the requirements of ICH Q6A for selection and could be used for quality control with better antitumor activity and pharmacokinetic data (unpublished data).

### 3.3. Crystal Structure Analysis of Solvatomorphs

The single crystal structures of Forms I, II and V were obtained and deposited in the Cambridge Crystal Structure Database. Despite repeated efforts, suitable crystals of Forms VIII and IX could not be obtained. Moreover, it was found that the simulated PXRD of three single crystals were in great agreement with the experimental PXRD result (Figures S13–S15). Details of the experimental crystal data are summarized in Table S7. The factors of R1 and wR2 were high because they mainly originated from the fractional occupational disorder of glucose groups in RA-XII, partial solvent disorder occupancy and induction by thermal vibration in the lattice [26]. For Form II, the non-integer stoichiometric ratio of H to O items in its formula might also be from the presence of multiple disordered water occupancy and glucose wagging [26]. This disorder was carefully modeled during the refinement process. Moreover, in three single-crystal structures, the skeleton of RA-XII was well-defined in the electron-density map. The core non-hydrogen atomic positions were reliably determined. The high residual factors were dominated by disordered solvent contributions. Therefore, the RA-XII skeleton conformation, hydrogen bonding pattern and crystal packing arrangement were accurately determined, and the core structural information was credible [27,28]. The SCXRD formula was not consistent with the TGA stoichiometry, which might be related to the form’s slightly hygroscopic property.

**Figure 9:**
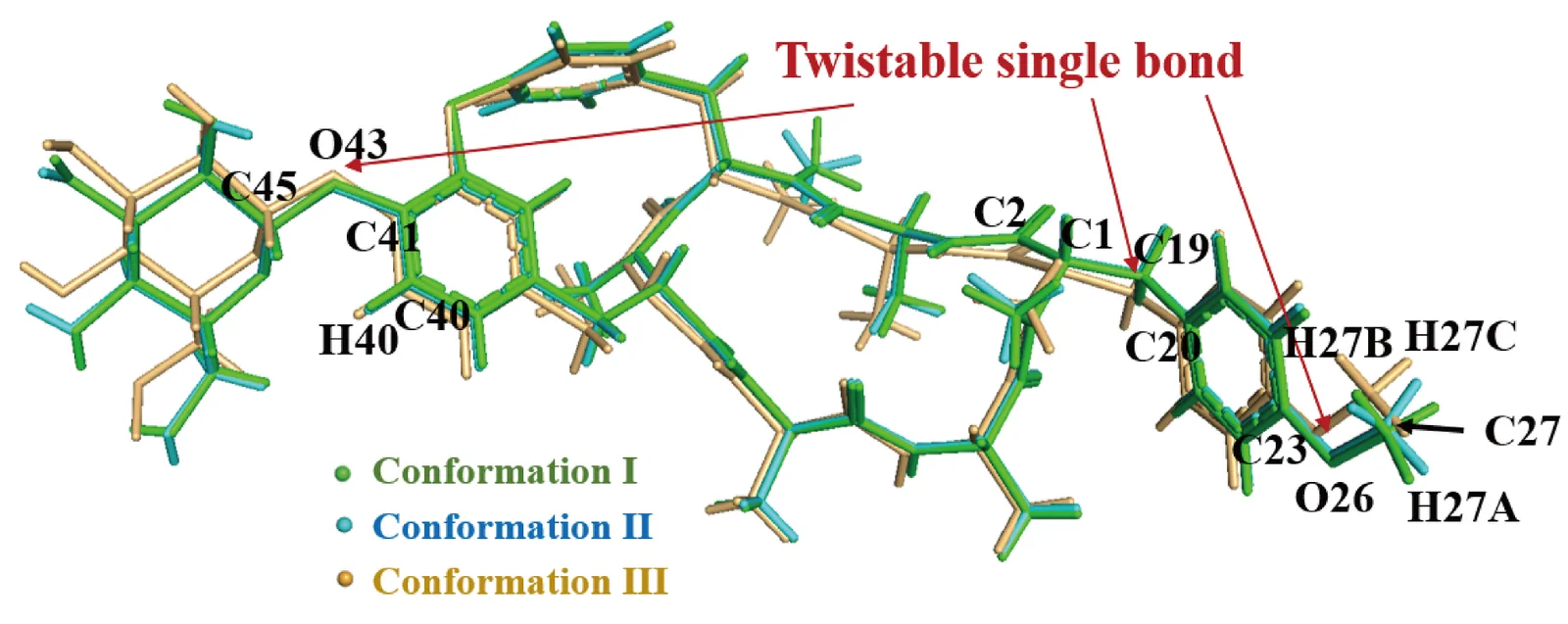
Overlay of three conformations in the crystal structures of RA-XII. The orange molecule represents Conformation I, green represents Conformation II, and blue represents Conformation III.

As is well- known, different conformations result in various polymorphs (conformational polymorphs), and different solvent-induced solvatomorphs can produce the same conformations due to interactions between solvents and API molecules [29,30,31,32,33]. As indicated by the literature [32], while conformations A and B had one or more torsion angles with Δθ > 95°, they were conformational polymorphs. Three forms (Forms I, II and V) contain one conformation. To distinguish among their conformational differences, they were identified as Conformations I, II and III, respectively. Their molecular conformations were stacked together to compare with one another (Figure 9). These conformations were primarily differentiated by six torsion angles, as presented in Table 4. The results indicated that Conformations I and II or III exhibit insignificant differences, with three Δθ > 95° (torsion angles of C23-O26-C27-H27A, C23-O26-C27-H27B and C23-O26-C27-H27C in Table 4). The differences resulted in the rotation of a single methoxy methyl group, which indicated that they were solvatomorphs with the same conformations; there was a little variation (all Δθ < 95°) between Conformations III (ethyl acetate) and II (water), which were solvatomorphs with the same conformation. Therefore, Conformations I and II or III represented the same conformation arising from different solvents or different solvent ratios, forming different solvatomorphs.

**Figure 10:**
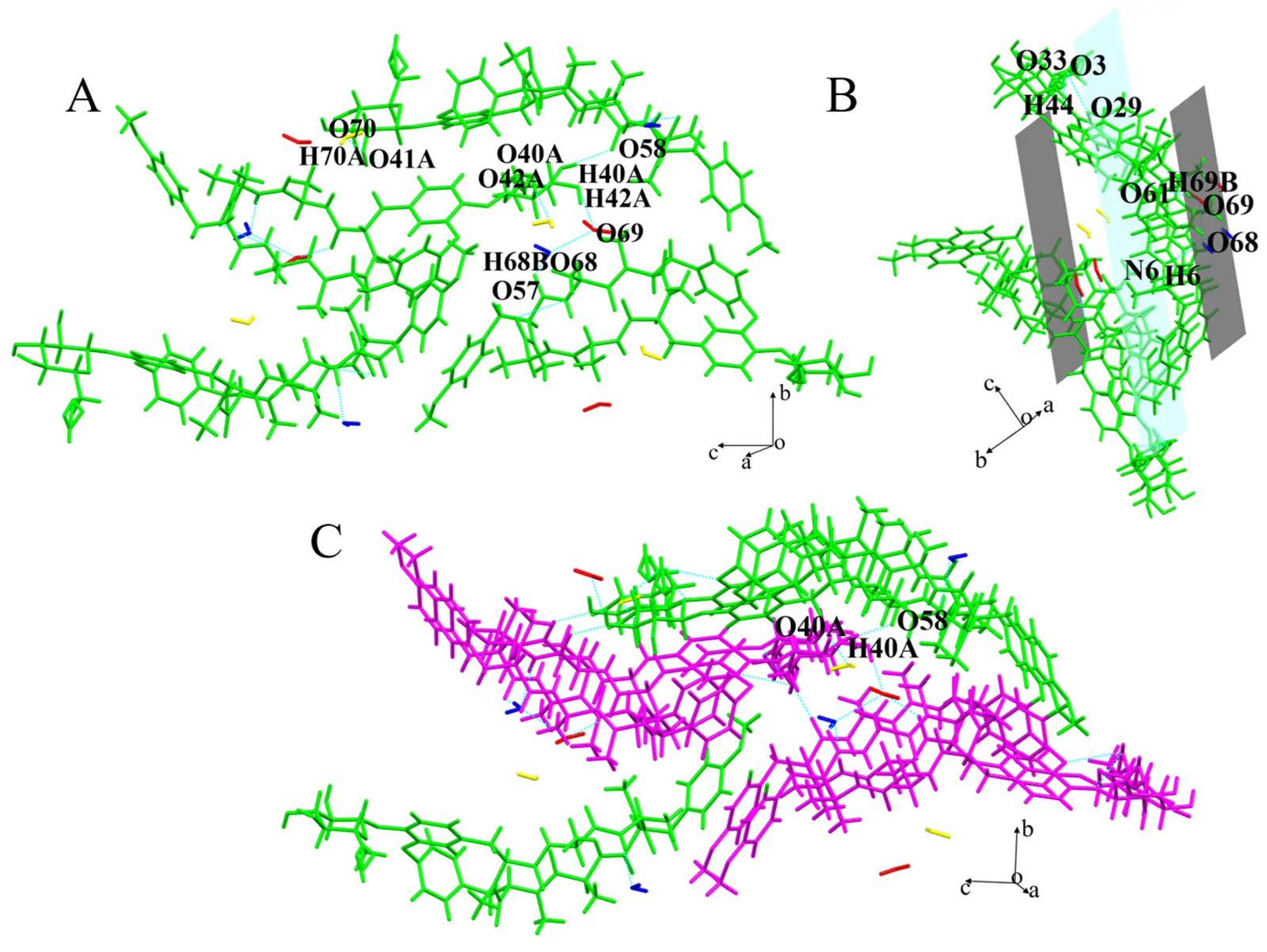
(**A**) One-dimensional chainlike structure, (**B**) 2D lattice-like structure, and (**C**) 3D spongy structure of Form I. Both colors represent Conformation I.

Form I also belonged to the orthorhombic system with the space group P2_1_2_1_2_1_. The amino, hydroxyl and carbonyl groups of RA-XII and H_2_O molecules were interconnected through N2−H2···O9 and O8−H8C···O6 hydrogen bonds to form dimers. The dimers were further linked with water molecules by O9−H9A···O2 and O48A−H48A···O8 hydrogen bonds, threading infinitely to form a chainlike structure, as depicted in Figure 10A and Table S8. The string structures were interconnected through water molecules with hydrogen bonds to form a 2D lattice-like structure, as shown in Figure 10B and Table S8. The 2D structure was fastened by intermolecular bonds, including O44A−H44D···O4 hydrogen bonds, and further linked with water molecules via the above hydrogen bonds to construct a 3D spongy structure in a mutually grid-parallel arrangement (Figure 10C and Table S8).

**Figure 11:**
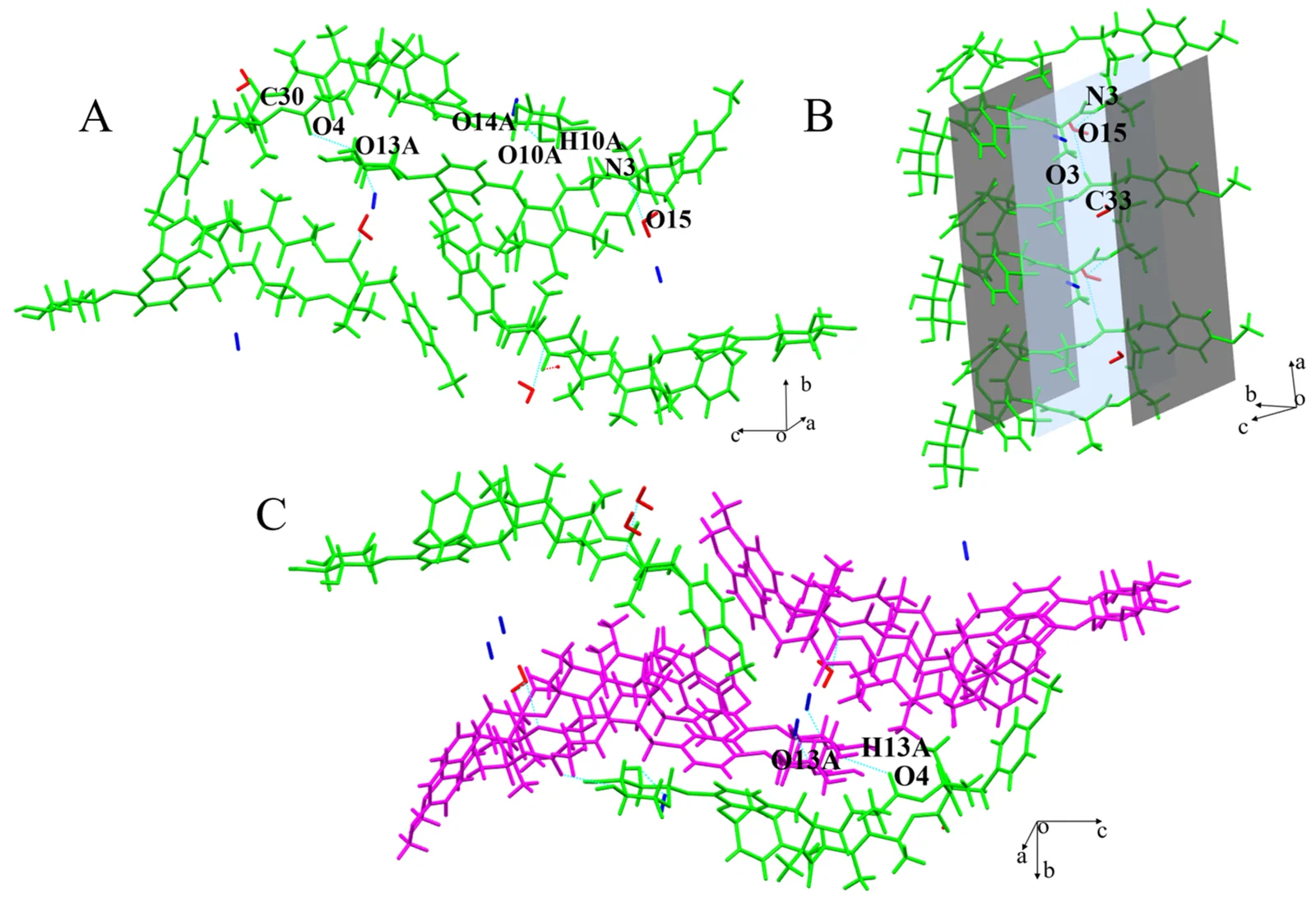
(**A**) One-dimensional chainlike structure, (**B**) 2D hierarchical structure, and (**C**) 3D layer structure of Form II. Both colors represent Conformation II.

Similarly to Form I, Form II belonged to the orthorhombic system with the space group P2_1_2_1_2_1_. The amino, hydroxyl and carbonyl groups of two RA-XII and one H_2_O molecule were interconnected through N3−H3B···O15, O10−H10A···O14A, and O13A−H13A···O4 hydrogen bonds, then further interconnected with two RA-XII molecules by the above hydrogen bonds, expanding infinitely to form chain structures, as depicted in Figure 11A and Table S9. The chain structures were linked via two water molecules with hydrogen bonds to form a 2D hierarchical structure, as depicted in Figure 11B and Table S9. These structures were linked with RA-XII and water molecules via O13A−H13A···O4 hydrogen bonds to construct a 3D layer-like structure that exhibited a layered arrangement (Figure 11C and Table S9).

**Figure 12:**
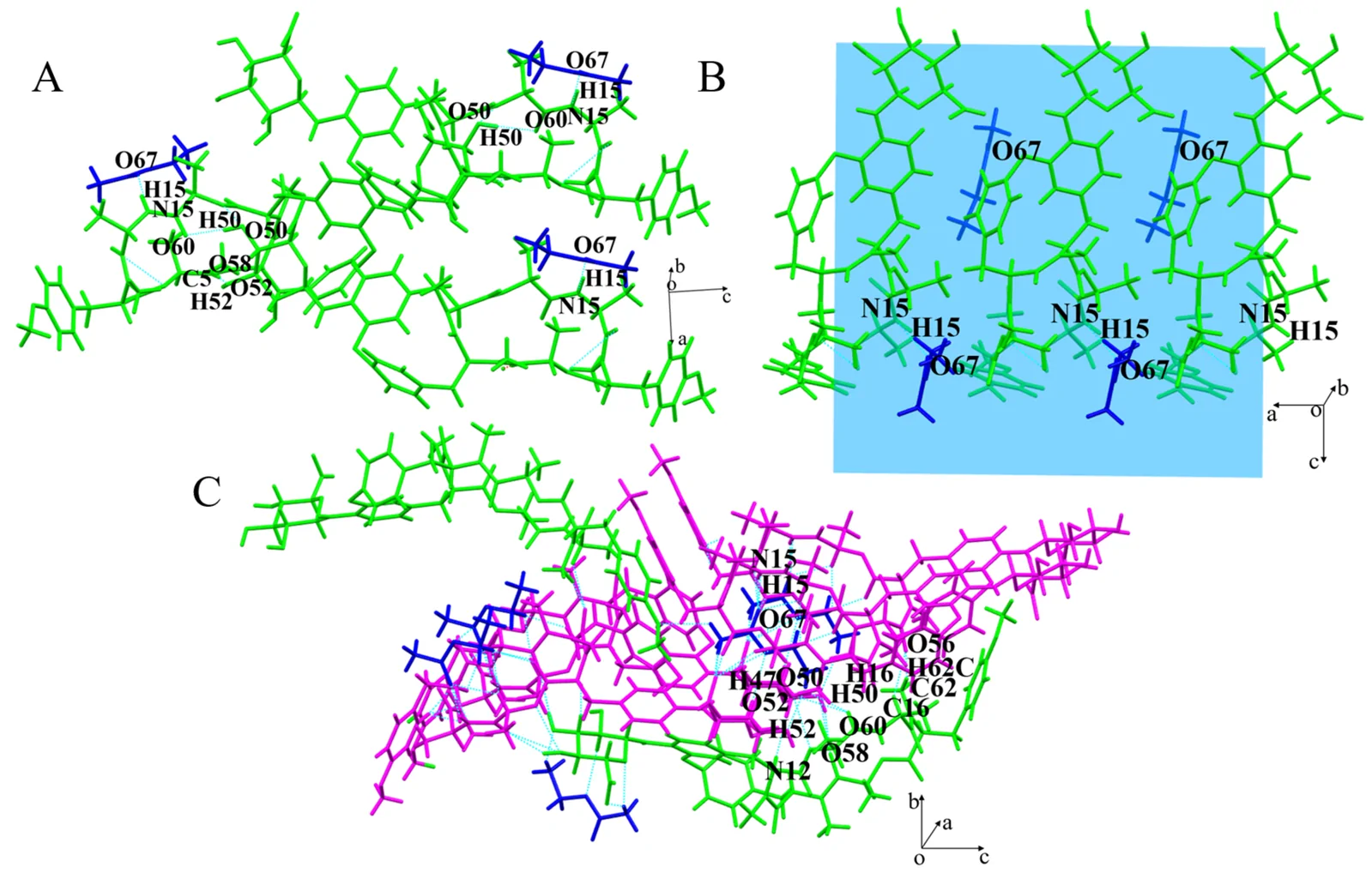
(**A**) Intermolecular hydrogen bonds, (**B**) 2D sheetlike structure, and (**C**) 3D tessellated brickwork structure of Form V. Both colors represent Conformation III.

Similarly, Form V contained Conformation III, which belonged to the orthorhombic system with the space group P2_1_2_1_2_1_. The amino, hydroxyl and carbonyl groups of two molecules were interconnected by hydrogen bonding, including O52−H52···O58 and O50−H50···O60, facilitating the formation of one-dimensional (1D) structures, as illustrated in Figure 12A and Table S10. The 1D structures were interconnected with ethyl acetate through N15−H15···O67 hydrogen bond, overlaying a 2D sheetlike structure, as shown in Figure 12B and Table S10. Then, these 2D structures were interconnected through O52−H52···O58, O50−H50···O60, C62−H62C···O56, and N12−H12···O50, and connected with an equimolar ratio of ethyl acetate by N15−H15···O67 hydrogen bonds, constructing a three-dimensional (3D) tessellated brickwork structure in a mutual cross-combination arrangement, as presented in Figure 12C and Table S10.

Moreover, as illustrated in Table S11, among the solvatomorphs, the lattice energy of Form I (−221.44 kJ/mol) was the lowest, combining the number of hydrogen bonds. The results indicated optimal intermolecular packing within its crystal lattice.

### 3.4. Hirshfeld Surface Analysis and Fingerprint Plots

**Figure 13:**
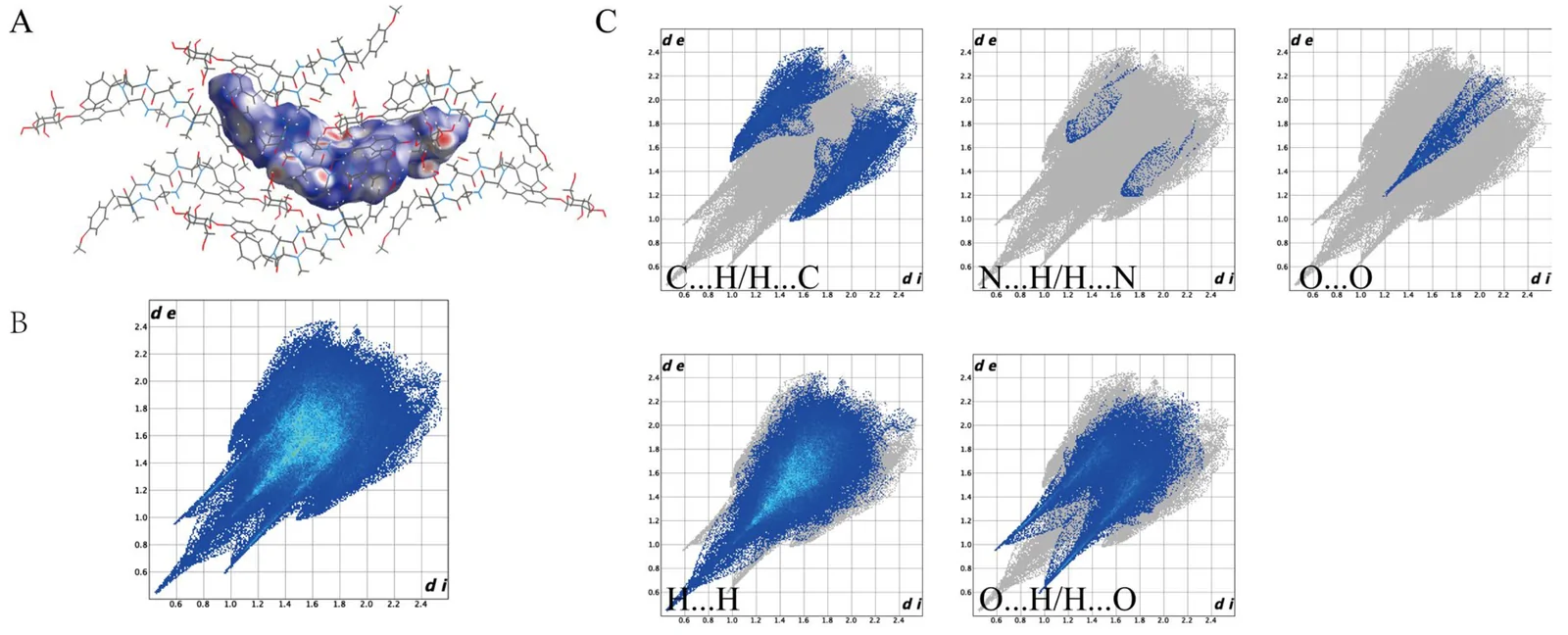
Hirshfeld surface maps (**A**) and fingerprint plots for Form I; (**B**) The total fingerprint maps; (**C**) The fingerprints are filtered by element type.

**Figure 14:**
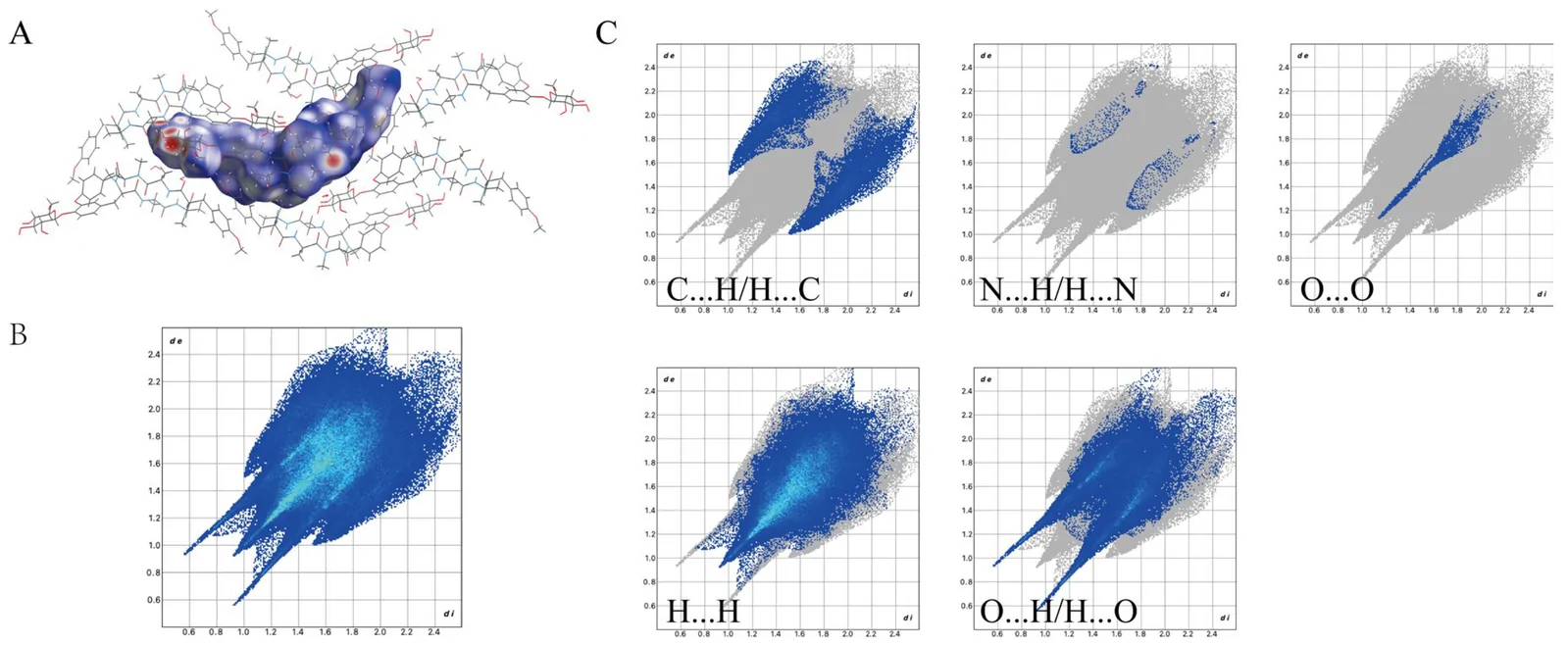
Hirshfeld surface maps (**A**) and fingerprint plots for Form II; (**B**) The total fingerprint maps; (**C**) The fingerprints are filtered by element type.

**Figure 15:**
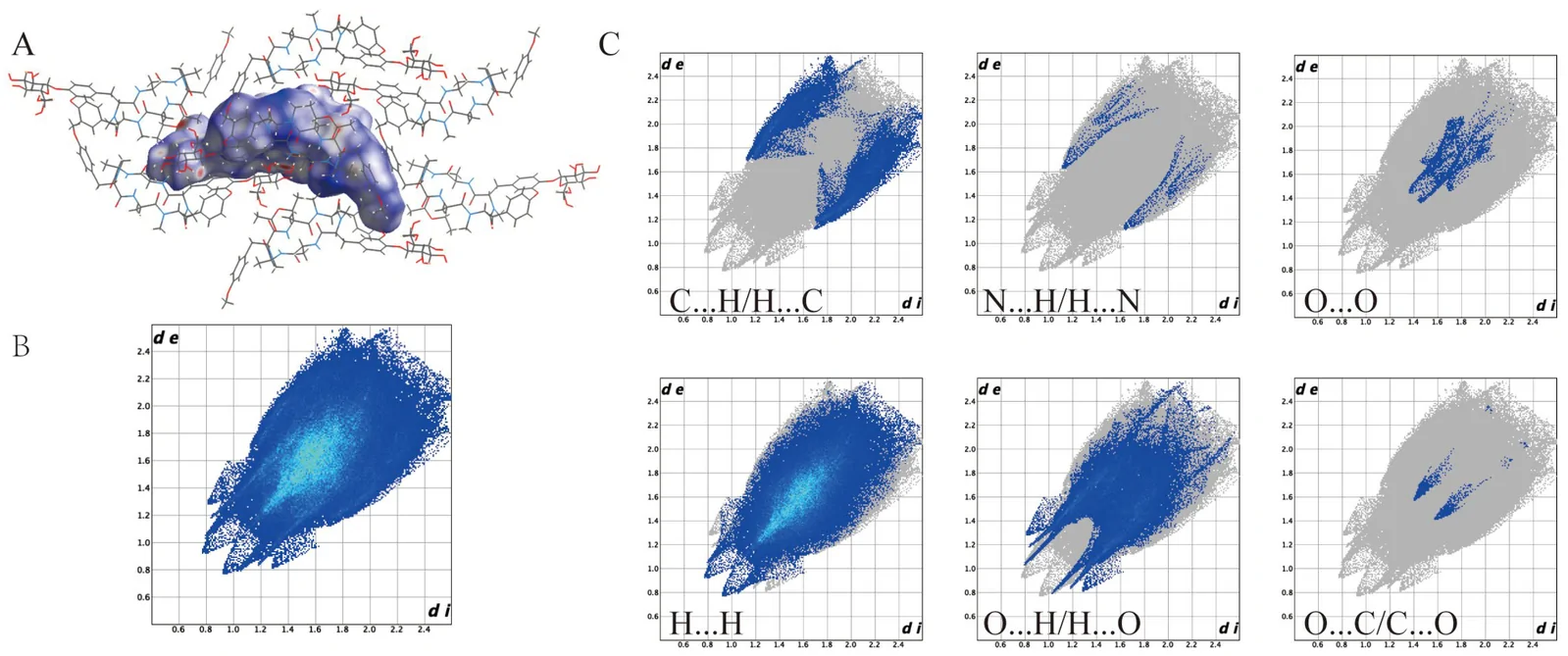
Hirshfeld surface maps (**A**) and fingerprint plots for Form V; (**B**) The total fingerprint maps; (**C**) The fingerprints are filtered by element type.

Hirshfeld surface analysis is a visual analysis method that offers underlying comprehension of intermolecular interactions among forms [34]. Hirshfeld surfaces of three RA-XII single crystals (Forms I, II and V) were calculated using Crystal Explorer 17.5. On Hirshfeld surface maps, the blue area indicated long contacts between atoms, while the red area represented the short contacts between atoms. The Hirshfeld surface maps for Forms I, II and V are illustrated in Figure 13A,B, Figure 14A,B and Figure 15A,B, respectively. The fingerprints are filtered by element type, as illustrated in Figure 13C, Figure 14C and Figure 15C.

**Figure 16:**
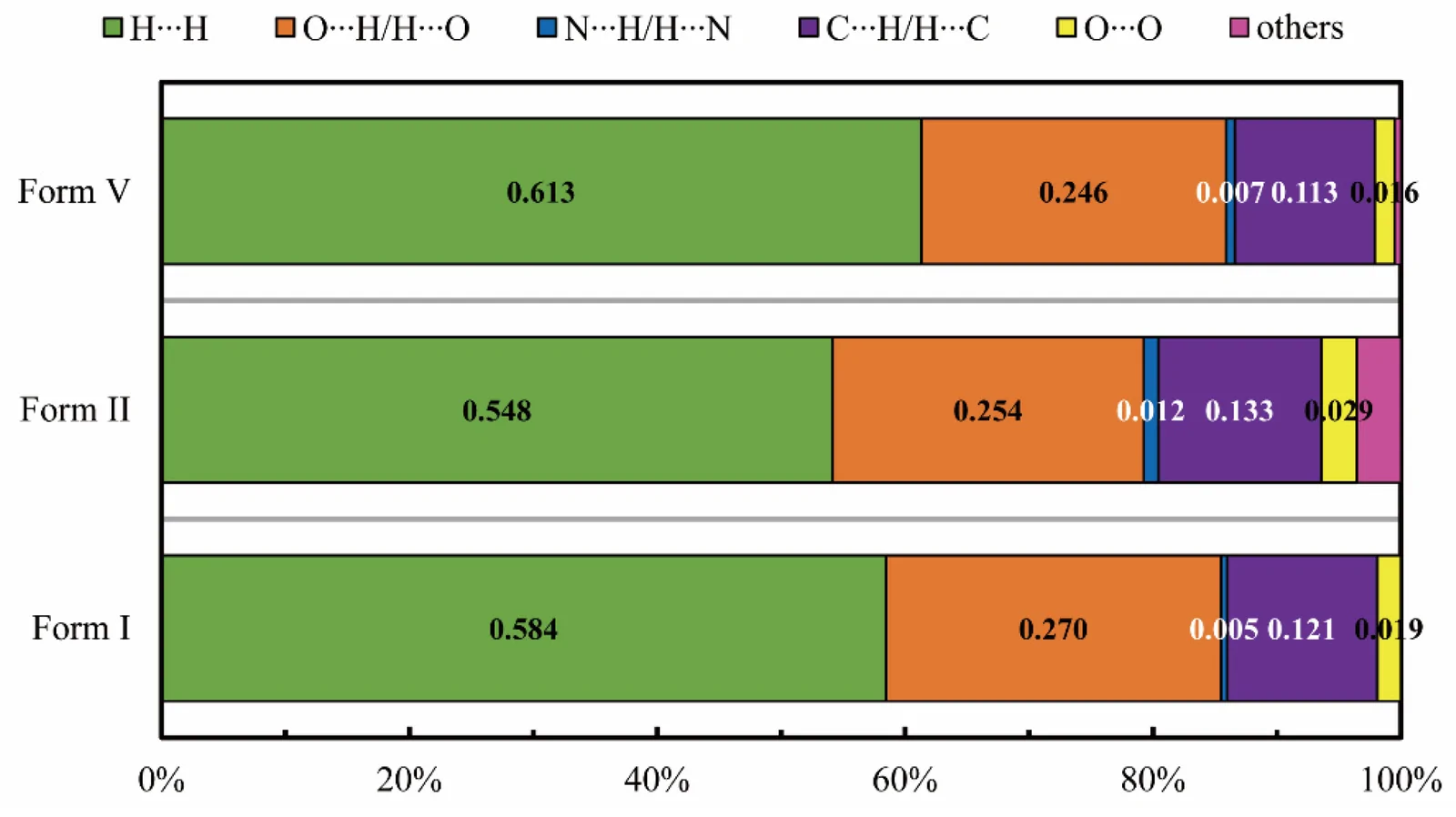
Relative contributions of different intermolecular contacts to the Hirshfeld surface areas.

The relative contributions of different interactions were calculated to compare the differences in hydrogen bonding of the forms, as illustrated in Figure 13C, Figure 14C, Figure 15C and Figure 16. H···H interactions occupied the largest proportion of crystal packing for the three forms, followed by O···H/H···O interactions, then C···H/H···C interactions. The O···H/H···O interactions, contributing 27.0% (Form I), 25.4% (Form II), and 24.6% (Form V) to the total surface contacts of crystal packing, respectively, demonstrated an indispensable role of intermolecular hydrogen bonding in the formation. Furthermore, the proportion of hydrogen bonds in Form V (O···H/H···O interactions) was less than that in the other two hydrates, showing the instability of Form V under high humidity, which was consistent with the structural analysis (Section 3.3) and the lower-humidity stability of Form V observed in Section 3.2. In conclusion, the fingerprint plots of the three forms showed slight differences, reflecting modest variations in hydrogen bonding in the RA-XII solvatomorphs that arise from distinct molecular packing modes.

### 3.5. Solute−Solvent Interactions in Different Solutions

**Figure 17:**
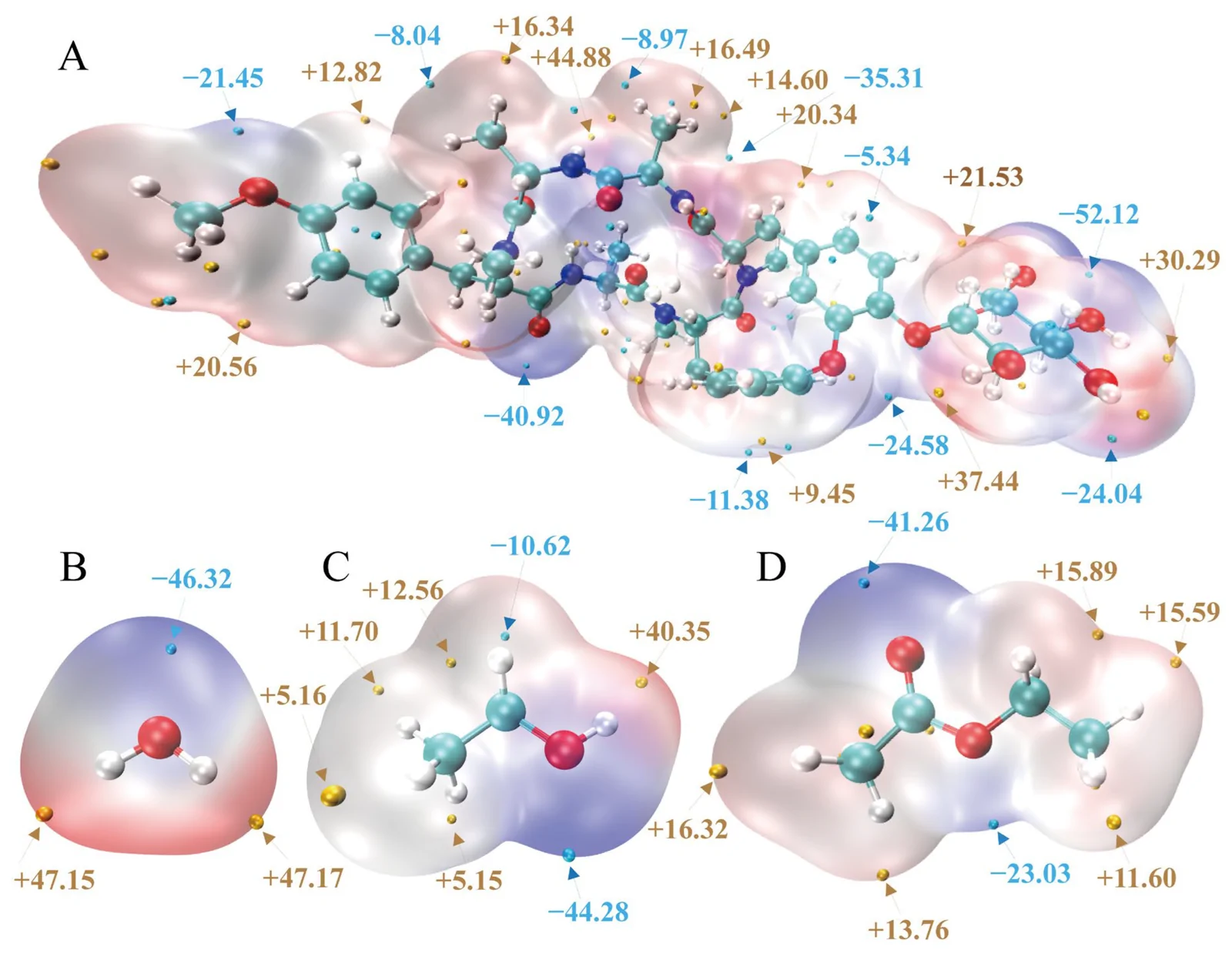
MEP surface of RA-XII and three solvents: (**A**) RA-XII, (**B**) water, (**C**) ethanol, and (**D**) ethyl acetate.

MEP surface, RDF, and solvation free energies were calculated to investigate molecular interactions in various solutions. The MEP surface is commonly used to depict the overall electronic distribution to assess intermolecular interactions [35]. The MEP surfaces of RA-XII and three solvent molecules are shown in Figure 17; the maximum and minimum of the electrostatic potential are marked with yellow and blue arrows, respectively. The electrostatic potential of the red area represents the negative nucleophilic attack zone where it is easier to give electrons or is more nucleophilic than other areas. And the positive potential of the blue area represents the positive electrophilic attack zone where it is easier to obtain electrons and is more electrophilic than other areas.

Firstly, the proton donor region and acceptor region of RA-XII molecules were gathered around the hydroxyl and carbonyl groups, respectively, marked with the blue areas in Figure 17, showing that the hydroxyl group of the glucose can both donate and accept protons to form hydrogen bonds. In addition, the oxygen atom of the water molecule exhibited obvious negative electrostatic potential, eventually exhibiting strong hydrogen bonding interactions with the solvent. However, the ester group of ethyl acetate showed relatively higher positive electrostatic potential values compared to those of water. These differences in electrostatic potential were consistent with the more-difficult-to-form crystals of Form V compared to hydrates, indicating hydrates formed more readily than ethyl acetates, which was consistent with some reported results [35,36]. In contrast with the water molecule, the hydroxyl group of ethanol presented higher positive electrostatic potential values, which might contribute to the formation of various hydrates.

**Figure 18:**
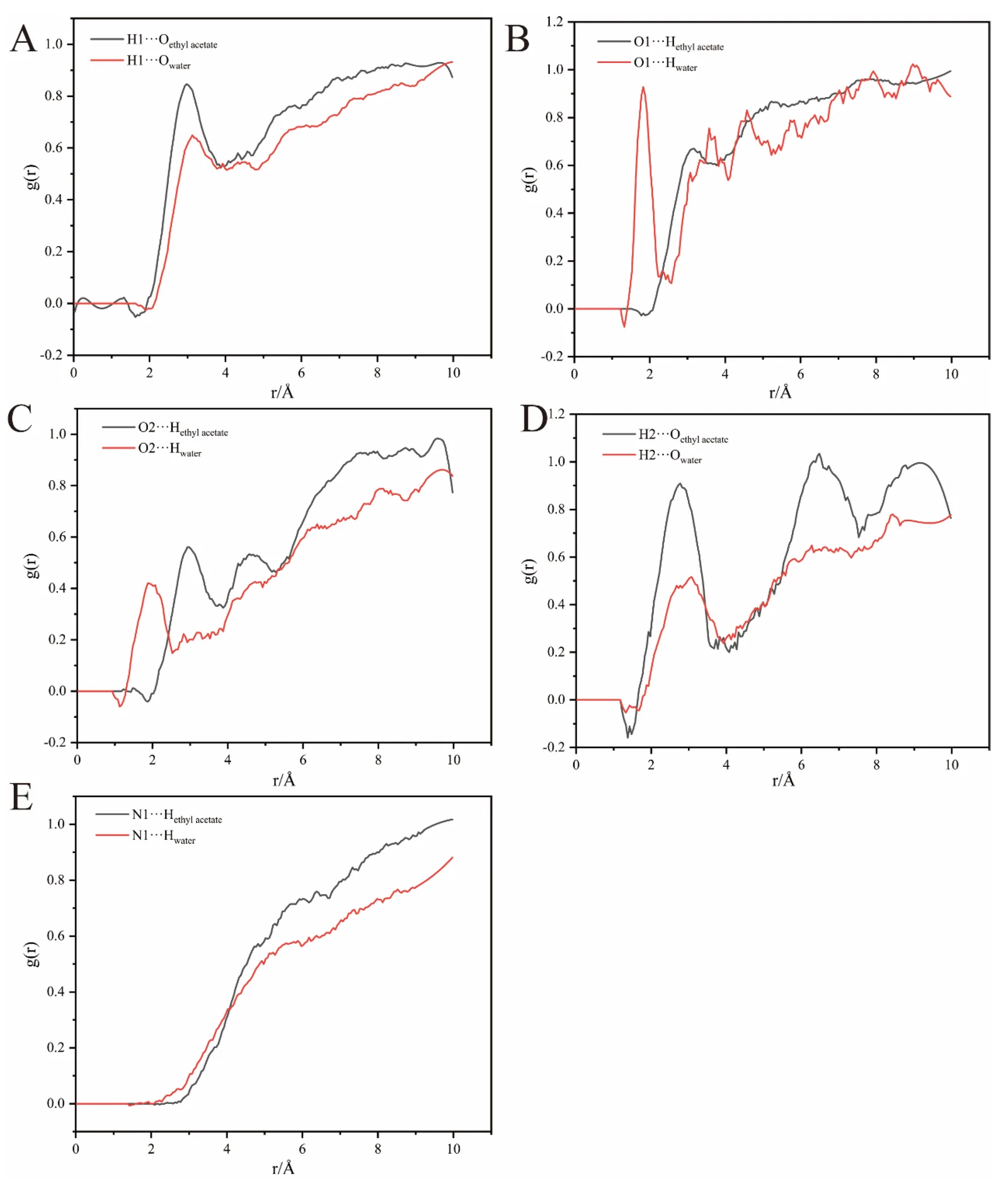
RDFs between the different solvent molecules and H and O atoms on the RA-XII molecule at 298.15 K: (**A**) H1 atom, (**B**) O1 atom, (**C**) O2 molecule, (**D**) H2 molecule, and (**E**) N1 atom.

MD simulations were conducted at 298.15 K to qualitatively measure the strength of hydrogen bonding interactions between RA-XII and two solvents. Based on their MEP surface analysis, RDF analysis was performed for the atoms that form hydrogen bonding interactions. According to the literature, the peak that appeared within 5 Å indicates the existence of a bond between two atoms; in particular, the peak located between 1.5 and 2.2 Å was typically recognized as indicative of hydrogen bonds [36,37]. As illustrated in Figure 18, H1 and O1 atoms were from the hydroxyl group; H2 and N1 were from the amino group, and the O2 molecule was from the carbonyl group. While RA-XII was dissolved in the ethanol-water system, strong peaks were observed for the O1 atoms in RA-XII and the H atom in the water molecule, suggesting strong hydrogen bonds between RA-XII and water, with RA-XII acting as a hydrogen donor. A weak peak was found for H2···O_water_, H1···O_water_ and O2···H_water_ on RA-XII. Considering the spatial structure of the RA-XII molecule, water molecules need to find an appropriate position to establish hydrogen bonds. Therefore, water molecules were more likely to interact with the O atom of RA-XII rather than with the H atom, especially in the –NH position of Alanine2, based on the analysis results for crystal structure. However, when RA-XII was dissolved in ethyl acetate, the H1 atom and H2 and O2 molecules in RA-XII and the O atom in the ethyl acetate molecule showed peaks indicating hydrogen bonds between the amino, carbonyl and hydroxyl groups of the RA-XII molecule and ethyl acetate. Meanwhile, the solvation free energies were computed to reflect the intrinsic molecular-solvent interaction strength of RA-XII in two solutions. As summarized in Table 5, the order of the solvation free energy was as follows: water < ethyl acetate. The interaction strength between RA-XII and water was significantly stronger than that with ethyl acetate, which was consistent with the analysis of the RDF. According to the crystal structure analysis, the O1 atoms and H2 molecules in RA-XII were more prone to interactions with two solvent molecules (Figure 18). Consequently, the simulation results indicated that the interaction strength of RA-XII with two solvents followed the order ethyl acetate < water, which might influence the difficulty of desolvation, ultimately affecting the stability of solvates. Therefore, the solvent was crucial for the formation of solvatomorphs.

### 3.6. Variable-Temperature PXRD of Form I Analysis

**Figure 19:**
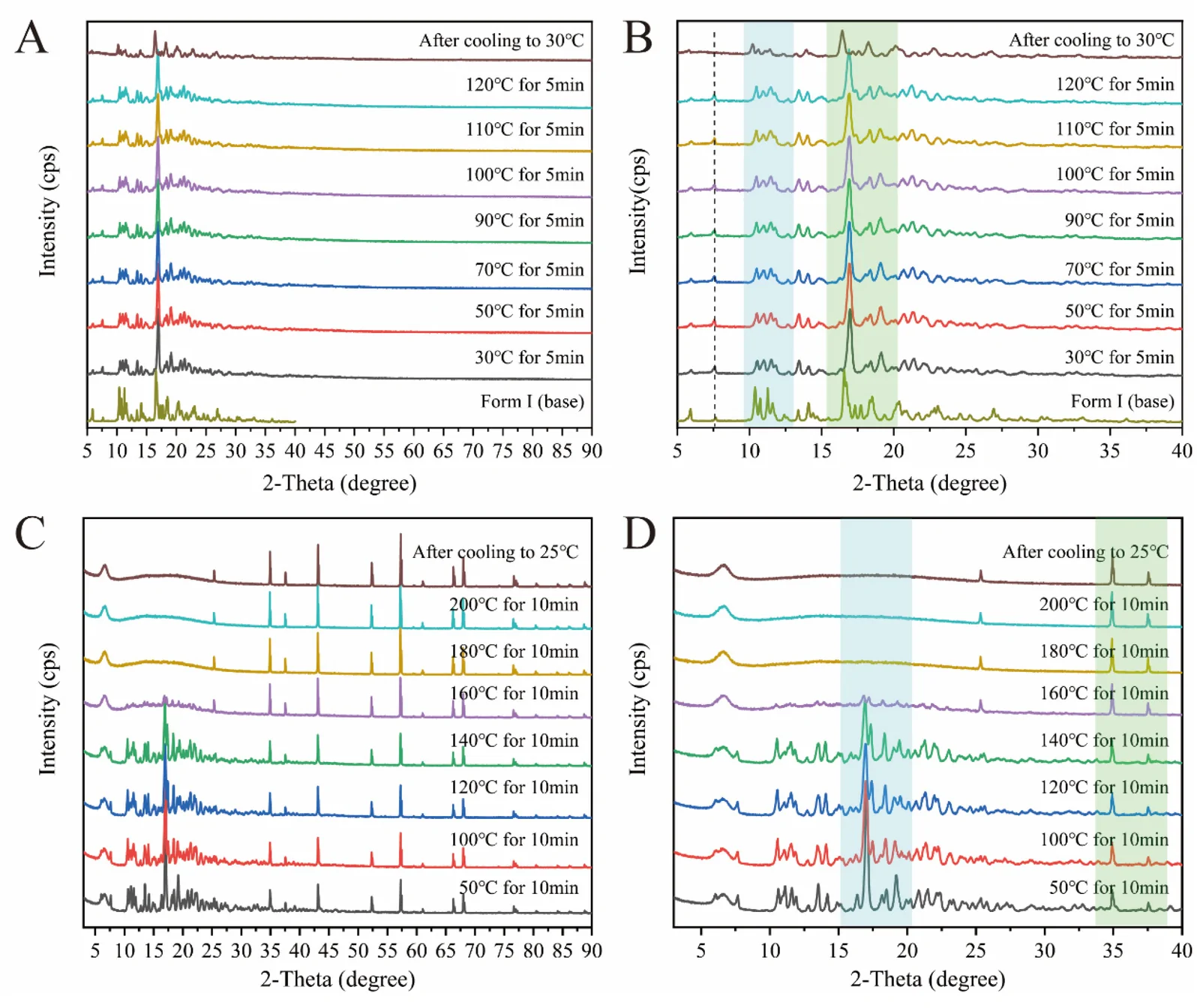
Variable-temperature PXRD profiles of the hydrates of Form I obtained at various temperatures ranging from 30 to 120 °C and held for 5 min: (**A**) overall (5–90°) and (**B**) magnified (5–40°). And variable-temperature PXRD profiles of the hydrates of Form I obtained at various temperatures ranging from 50 to 200 °C and held for 10 min: (**C**) overall (5–90°) and (**D**) magnified (5–40°).

The potential crystallinity transformation of the hydrates of Form I was studied with variable-temperature PXRD, which was performed at controlled temperature to remove the existence of pseudo-polymorphs between anhydrate and hydrate [38], as illustrated in Figure 19. Form I (base form) was heated from 30 to 120 °C at a rate of 10 °C/min and held for 5 min at seven heat points (30 °C, 50 °C, 70 °C, 90 °C, 100 °C, 110 °C, 120 °C) and 10 min at seven heat points (50 °C, 100 °C, 120 °C, 140 °C, 160 °C, 180 °C, 200 °C). The entire process was conducted under a vacuum state. Form I was transformed into another form under the measured conditions from 30 °C to 120 °C, particularly the temperature up to 160 °C. The PXRD diffraction peak differences are illustrated in the green areas of Figure 19A,B. To further explore the importance of the hydrates–anhydrous relationship, Form I was placed in a vacuum oven and dried for 4 h at 110 °C to ensure that the crystalline water was removed. Then, PXRD, DSC, and TGA characterizations of the resulting dried Form I were performed. The results are shown in Figures S16 and S17 in the supporting information. The PXRD results indicated that the diffraction peaks for Form I before and after drying were consistent, demonstrating that Form I transition did not occur during the drying process. The TG-DSC results showed the dried Form I did not exhibit weight loss corresponding to solvent desorption prior to melting at 212.85 °C. The exothermic peak at 249.92 °C on the DSC curve was attributed to cold crystallization of the molten state. The above results indicated that Form I was a channel-type solvate: water molecules are present in continuous channels within the lattice, and the RA-XII molecular stacking framework does not collapse after the solvent is removed by drying. The result was different from that of the DSC characterization: the dehydrate melting point of Form I was 251.56 °C—a value 39 °C higher, which was closer to the new cold crystallization exotherm. Upon removal of water, Form I transformed into a new dehydrated phase. The endotherm at 212.85 °C is the melting of this dehydrated form. The subsequent exothermic event at 249.92 °C is assigned to recrystallization from the molten state, not melting. Although the PXRD patterns remained consistent before and after drying Form I, this might be related to moisture absorption by Form I after drying. The results indicated that the 251.56 °C event in the original DSC trace may correspond to a melt–recrystallisation–remelt sequence rather than a single fusion event. They were not the same solid phase, which was different from the results of VT-PXRD. When kept at temperatures below 160 °C in a vacuum, Form I could still be observed after cooling to 25 °C; however, when the temperature exceeded 160 °C and the form was cooled down to 25 °C, reabsorption of water cannot lead to regeneration of the original base form, but to the amorphous and other forms as depicted in Figure 19C,D. The results were consistent with that observed after drying at 110 °C for 4 h. Further results of HPLC determination indicated that the deglycosylation of RA-XII occurred within a temperature range of 50–200 °C, maintained for 10 min throughout the process (Figure S18). This indicates that, based on the observations and results from DSC and VT-PXRD experiments (Figure 19D), prolonged processing of the API (Form I) at temperatures exceeding 60 °C should be avoided when it is used in the pharmaceutical manufacturing process, and that the API (Form I) should be kept in a well-ventilated environment at low temperature during its preparation and storage.

## 4. Conclusions

In this study, the structures and formation mechanisms of RA-XII solvatomorphs were explored through both experimental investigations and molecular dynamics simulations. Six forms (Forms I, II, III, V, VIII and IX) were comprehensively investigated, with PXRD, thermal (DSC, TGA), spectroscopy (FT-IR, RM), morphology (MP) and SEM analyses used to characterize the solvatomorphs and amorphous form. Single crystals of Forms I, II and V were successfully obtained for the first time, and their conformations and molecular packing structures were analyzed. The results indicated that Form I was physically more stable and exhibited good aqueous solubility. Single-crystal structures and Hirshfeld surface analysis indicated that the proportion of hydrogen bonding interactions between O and H atoms and molecular packing were significantly different among the RA-XII solvatomorphs. Furthermore, MEP surface analysis and molecular dynamics simulations suggested that the hydrogen bonding interaction strengths between RA-XII and solvents were as follows: ethyl acetate < water, influencing the solvation feasibility and stability of the forms and consequently generating solvatomorphs. These findings are valuable for understanding the solvatomorph formation mechanisms and quality control of RA-XII as an anti-tumor API.

## Figures and Tables

**Table 1:** Melting temperatures and enthalpies of fusion of RA-XII solvatomorphs and amorphous form.

Forms	I	II	III	V	VIII	IX
Melting temperature T_m_ (°C)	251.56	246.95	-	254.28	268.47	266.44
ΔH_fus_ (J/g)	18.79	28.16	-	22.68	38.80	38.27

**Table 2:** FT-IR spectra vibrations of six forms of RA-XII.

Forms	C=O Stretching Vibration (cm^−1^)	O–H Stretching Vibration (cm^−1^)	C–H Out of Plane Bending Vibration (cm^−1^)	N–H Out of Plane Bending Vibration (cm^−1^)	C–O Stretching Vibration (cm^−1^)
I	1657	3416, 3568	2930, 2984	3061, 3046	1265, 1242, 1211
II	1659	3416, 3568	2930, 2982	3063, 3038	1263, 1242, 1213
III	1661	3414	2934, 3009	3059	1248, 1213
V	1707, 1663	3414, 3298	2932, 2980	3298, 3061	1254, 1217
VIII	1665	3566, 3418	2930, 2982	3061, 3040	1261, 1242, 1213
IX	1663	3416,3566	2930, 2982	3065, 3042	1263, 1244, 1213

**Table 3:** Characteristic peaks in RM patterns of six RA-XII forms.

Forms	Characteristic Peaks
Form I	133.52, 1207.6, 2931.83, 3061.85
Form II	2931.83, 3061.85
Form III	1209.20, 1610.38, 2935.52, 3065.45
Form V	129.76, 800.59, 862.16, 1177.32, 1206.02, 1301.00, 1654.00, 2930.60, 2982.05, 3010.01, 3069.04
Form VIII	129.76, 1206.02, 2931.83, 2983.27, 3061.85
Form IX	131.64, 1207.60, 1605.96, 2931.83, 2982.05, 3010.01, 3063.05

**Table 4:** Torsion angles (τ, deg) of three conformations of RA-XII solvatomorphs.

Torsion Angle	Conformation I (Form I)	Conformation II (Form II)	Conformation III (Form V)
C2-C1-C19-C20	−163.04	−162.52	−166.33
O43-C41-C40-H40	−6.27	−5.23	−0.38
C45-O43-C41-C40	−4.75	−5.84	−11.68
C23-O26-C27-H27A	163.40	−173.16	−175.99
C23-O26-C27-H27B	−76.60	66.84	64.01
C23-O26-C27-H27C	43.40	−53.16	−55.99

**Table 5:** Solvation free energy in the two solvents (values in kcal·mol^−1^).

Solvent	ΔGid	ΔGvdw	ΔGelec	ΔGsolv
Water	18.656	76.579	−507.151	−411.916
Ethyl acetate	72.213	188.358	−78.626	181.945

## Data Availability

The CIF files obtained from single-crystal X-ray diffraction are available from the Cambridge Crystallographic Data Centre: CCDC numbers 2529513 (Form I), 2529518 (Form II) and 2529516 (Form V). The other raw data presented in this article will be made available upon request.

## Supplementary Materials

The following supporting information can be downloaded at: https://www.mdpi.com/article/10.3390/pharmaceutics18101285/s1, Figure S1: PXRD results of stability pre-experiments for three forms for 10 days: (A) Form IV, (B) Form VI, (C) Form VII; Figure S2: FT-IR spectra of Form IV, VI and VII; Figure S3: Characterization of three RA-XII solvatomorphs. TGA (blue lines) and DSC (red lines) analyses for (A) Form IV, (B) Form VI, (C) Form VII; Figure S4: Microscopy images of (A) Form IV, (B) Form VI, (C) Form VII; Figure S5: Concentration solubility of six RA-XII forms in water from dynamics of crystallization; Figure S6: The linear relationship between ln(x) and 1/T for six API forms in an aqueous system; Figure S7: Equilibrium solubility of six RA-XII forms; Figure S8: Dissolution kinetics curves of six RA-XII forms in distilled water; Figure S9: Dissolution kinetics curves of six RA-XII forms in pH 7.4 PBS solution; Figure S10: Solubility of six RA-XII forms in different pH buffer solutions; Figure S11: Hygroscopicity study of six RA-XII forms at 25 °C and 90% RH; Figure S12: Characteristic PXRD patterns of RA-XII gram-scaled Form I; Figure S13: Calculated and experimental PXRD patterns of Form I; Figure S14: Calculated and experimental PXRD patterns of Form II; Figure S15: Calculated and experimental PXRD patterns of Form V; Figure S16: Characteristic PXRD patterns of RA-XII Form I after drying and before drying; Figure S17: Characterization of RA-XII Form I after drying. TGA (blue lines) and DSC (red lines) analyses: Figure S18: HPLC chromatograms of hydrate (Form I) after measured variable-temperature PXRD profile; Table S1: Preparation methods for various solvatomorphs and amorphous form of RA-XII; Table S2: The 2-Theta in PXRD patterns of RA-XII solvatomorphs and amorphous form; Table S3: Purity results of stability pre-experiments for three RA-XII solvatomorphs (Forms IV, VI and VII); Table S4: The parameters of six API forms in water system; Table S5: The equilibrium solubility data of six RA-XII forms; Table S6: Dissolution curves and fitting equations of six API forms in water and pH 7.4 PBS; Table S7: Crystal structure parameters of three RA-XII single crystals; Table S8: Hydrogen bonds in crystal structures of conformation I (Form I); Table S9: Hydrogen bonds in crystal structures of conformation II (Form II); Table S10: Hydrogen bonds in crystal structures of conformation III (Form V); Table S11: Summary of the lattice energies.

## References

[1] Hossain Mithu M.S., Economidou S., Trivedi V., Bhatt S., Douroumis D. (2021). Advanced methodologies for pharmaceutical salt synthesis. Cryst. Growth Des..

[2] Shalaev E., Wu K., Shamblin S., Krzyzaniak J.F., Descamps M. (2016). Crystalline mesophases: Structure, mobility, and pharmaceutical properties. Adv. Drug Deliv. Rev..

[3] Shi Q., Chen H.B., Wang Y.N., Xu J., Liu Z.Y., Zhang C. (2022). Recent advances in drug polymorphs: Aspects of pharmaceutical properties and selective crystallization. Int. J. Pharm..

[4] Zhang T.T., Yang S.Y., Zhang B.X., Yang D.Z., Lu Y., Du G.H. (2022). Insights into the properties of Amygdalin solvatomorphs: X-ray structures, intermolecular interactions, and transformations. ACS Omega.

[5] Braga D., Casali L., Grepioni F. (2022). The relevance of crystal forms in the pharmaceutical field: Sword of damocles or innovation tools?. Int. J. Mol. Sci..

[6] Li W.L., Zhou L.N., Tian B.Q., Chen K., Feng Y.G., Wang T., Wang N., Huang X., Hao H.X. (2023). Polymorphism of pradofloxacin: Crystal structure analysis, stability study, and phase transformation behavior. Pharm. Res..

[7] Thakral N.K., Thakral S., Stephenson G.A., Sedlock R., Suryanarayanan R. (2019). Compression-induced polymorphic transformation in tablets: Role of shear stress and development of mitigation strategies. J. Pharm. Sci..

[8] Lin Q.W., Liu M.Y., Yue G.G.L., Cheung M.K., Lai Z.X., Kwok F.H.F., Lee J.K.M., Wang Z., Lau C.B.S., Tan N.H. (2022). Anti-inflammatory activities of natural cyclopeptide RA-XII in colitis-associated colon cancer mouse model and its effect on gut microbiome. Phyther. Res..

[9] Wang J., Wang J., Li L., Feng L., Wang Y.R., Wang Z., Tan N.H. (2021). RA-XII, a bicyclic hexapeptidic glucoside isolated from *Rubia yunnanensis* Diels, exerts antitumor activity by inhibiting protective autophagy and activating Akt-mTOR pathway in colorectal cancer cells. J. Ethnopharmacol..

[10] Wang Y.R., Guo D., He J.Q., Song L.H., Chen H.C., Zhang Z.H., Tan N.H. (2019). Inhibition of fatty acid synthesis arrests colorectal neoplasm growth and metastasis: Anti-cancer therapeutical effects of natural cyclopeptide RA-XII. Biochem. Biophys. Res. Commun..

[11] Liu M.Y., Lai Z.X., Zhu L.J., Ding X., Tong X.Y., Wang Z., Bi Q.R., Tan N.H. (2021). Novel amorphous solid dispersion based on natural deep eutectic solvent for enhancing delivery of anti-tumor RA-XII by oral administration in rats. Eur. J. Pharm. Sci..

[12] Xu Y.Q., Yao Y.R., Wang L.X., Chen H.C., Tan N.H. (2021). Hyaluronic acid coated liposomes co-delivery of natural cyclic peptide RA-XII and mitochondrial targeted photosensitizer for highly selective precise combined treatment of colon cancer. Int. J. Nanomed..

[13] Spackman P.R., Turner M.J., McKinnon J.J., Wolff S.K., Grimwood D.J., Jayatilaka D., Spackman M.A. (2021). CrystalExplorer: A program for Hirshfeld surface analysis, visualization and quantitative analysis of molecular crystals. J. Appl. Crystallogr..

[14] Stratmann R.E., Burant J.C., Scuseria G.E., Frisch M.J. (1997). Improving harmonic vibrational frequencies calculations in density functional theory. J. Chem. Phys..

[15] Mazurek A.H., Szeleszczuk Ł., Pisklak D.M. (2020). Periodic DFT calculations—Review of applications in the pharmaceutical sciences. Pharmaceutics.

[16] Humphrey W., Dalke A., Schulten K. (1996). VMD: Visual molecular dynamics. J. Mol. Graph..

[17] Lu T., Chen F.W. (2012). Multiwfn: A multifunctional wavefunction analyzer. J. Comput. Chem..

[18] Sun H., Jin Z., Yang C.W., Akkermans R.L.C., Robertson S.H., Spenley N.A., Miller S., Todd S.M. (2016). COMPASS II: Extended coverage for polymer and drug-like molecule databases. J. Mol. Model..

[19] Burger A., Ramberger R. (1979). On the polymorphism of pharmaceuticals and other molecular crystals. I. Mikrochim Acta.

[20] Moyo D.S., van der Merwe E.M., Rademeyer M., Malan F.P., Atanasova M.T., Mapossa A.B., Focke W.W. (2023). Characterizing the thermal phase behaviour of fipronil polymorphs. J. Therm. Anal. Calorim..

[21] Feliciano I.O., Bernardes C.E.S., Piedade M.F.M., Santos M.S.C.S., Blahut J., Dračínský M., Minas da Piedade M.E. (2026). New insights into the structure and thermodynamic stability of polymorphs I and II of the nicotinamide: Adipic acid co-crystal: A BEST-CSP study. Cryst. Growth Des..

[22] Thomas S., Dougay J.A., Annunziata O. (2025). Yield of protein crystallization from metastable liquid-liquid phase separation. Molecules.

[23] Ilevbare G.A., Taylor L.S. (2013). Liquid-liquid phase separation in highly supersaturated aqueous solutions of poorly water-soluble drugs: Implications for solubility enhancing formulations. Cryst. Growth Des..

[24] Urbanova M., Sturcova A., Kredatusova J., Brus J. (2015). Structural insight into the physical stability of amorphous simvastatin dispersed in pHPMA: Enhanced dynamics and local clustering as evidenced by solid-state NMR and Raman spectroscopy. Int. J. Pharm..

[25] Reutzel-Edens S.M., Kleemann R.L., Lewellen P.L., Borghese A.L., Antoine L.J. (2003). Crystal forms of LY334370 HCl: Isolation, solid-state characterization, and physicochemical properties. J. Pharm. Sci..

[26] Tamás Gál G., May N.V., Trif L., Mihály J., Bombicz P. (2021). Solvatomorph and polymorph screening of clopamide drug and its copper (II) complex crystals. CrystEngComm.

[27] Pindelska E., Marczewska-Rak A., Jaśkowska J., Madura I.D. (2021). Solvates of new arylpiperazine salicylamide derivative-a multi-technique approach to the description of 5 HTR ligand structure and interactions. Int. J. Mol. Sci..

[28] Lin F.X., Qiu B.Q., Lou B.Y. (2017). Preparation, crystal structure and solubility of triethylamine salt monohydrate of naringenin. Chin. J. Appl. Chem..

[29] Wu H.C., Wang T., Wu D., Feng Y.G., Yao Y.T., Zhang J.M., Zhang Y.F., Huang X., Wang N., Hao H.X. (2024). Insight into the structure and formation mechanism of esmolol hydrochloride polymorphs based on experiments and molecular simulations. Cryst. Growth Des..

[30] Aitipamula S., Chow P.S., Tan R.B.H. (2010). Conformational and enantiotropic polymorphism of a 1:1 cocrystal involving ethenzamide and ethylmalonic acid. CrystEngComm.

[31] Braun D.E., Gelbrich T., Kahlenberg V., Tessadri R., Wieser J., Griesser U.J. (2009). Conformational polymorphism in aripiprazole: Preparation, stability and structure of five modifications. J. Pharm. Sci..

[32] Cruz-Cabeza A.J., Bernstein J. (2014). Conformational polymorphism. Chem. Rev..

[33] Zhang X.R., He S.F., Zhang S., Li J., Li S., Liu J.S., Zhang L. (2017). Polymorphs and solvatomorphs of azilsartan medoxomil: Elucidation of solvent-induced construction and conformational diversity. J. Mol. Struct..

[34] Vasconcelos S.A., Tenorio J.C., Gurgel J.V.M., Benevides C.C., Nazario C.E.D., Carvalho P.S. (2025). Polymorphism of racemic (±)-mefloquine free base: The role of enantiomeric recognition in polymorph assemblies. J. Mol. Struct..

[35] Liu C.H., Wang Y.L., Zhao Y.H., Li X., Wang N., Huang X., Wang T., Hao H.X. (2022). Selective nucleation of polymorphs induced by multiple conformations and self-association. Cryst. Growth Des..

[36] Xu Y.C., Tang S., Li Z.Q., Liang W.D., Xu J.K., Ouyang J.B., Zhang C.T. (2023). Uncovering the solvent-solute interaction mechanism in nucleation of erythritol based on metastable zone widths: Experimental kinetics and molecular dynamics simulations. Ind. Eng. Chem. Res..

[37] Wen T., Wang H.R., Li C.R., Xu J.K., Zhang C.T. (2022). Insight into the role of hydrochloric acid in the thermodynamics and nucleation kinetics behavior of arbidol hydrochloride monohydrate from metastable zone widths. CrystEngComm.

[38] Miyano T., Sugita K., Ueda H. (2024). The continuous and reversible transformation of the polymorphs of an MGAT2 inhibitor (S-309309) from the anhydrate to the hydrate in response to relative humidity. Pharmaceutics.

