# Supplementary material for: Preparation and Investigation of Solvatomorphs of Anti-Tumor Candidate RA-XII
Source: Pharmaceutics. 2026 Oct 10;18(10):1285. doi: 10.3390/pharmaceutics18101285 (PMC13708435; doi:10.3390/pharmaceutics18101285)
Supplement: Supplementary file 1 [file pharmaceutics-18-01285-s001.zip › pharmaceutics-4294316-supplementary.pdf]

Supporting Information

Preparation and investigation of anti-tumor candidate RA-XII solvatomorphs

Feng Cao[†], Song Zhang[†], Qicheng Duan, Haitao Dou, Yinghui Liang and Ninghua Tan*

Authors: Department of TCMs Pharmaceuticals, School of Traditional Chinese Pharmacy, China Pharmaceutical University, Nanjing 211198, China.

*Corresponding Author: Ninghua Tan, Department of TCMs Pharmaceuticals, School of Traditional Chinese Pharmacy, China Pharmaceutical University, Nanjing 211198, People's Republic of China.

Catalog

Figure S1. PXRD results of stability pre-experiments of three forms for 10 days 3
Figure S2. FT-IR spectra of Form IV, VI and VII 3
Figure S3. Characterization of three RA-XII solvatomorphs....4
Figure S4. Microscopy images of (A) Form IV, (B) Form VI, (C) Form VII 4
Figure S5. Concentration solubility of six RA-XII forms in water....5
Figure S6. The linear relationship between ln(x) and 1/T for 6 API forms in an aqueous system....5
Figure S7. Equilibrium solubility of six RA-XII forms....6
Figure S8. Dissolution kinetics curves of six RA-XII forms in distilled water 6
Figure S9. Dissolution kinetics curves of six RA-XII forms in pH 7.4 PBS solution 7
Figure S10. Solubility of six RA-XII forms in different pH buffer solutions....7
Figure S11. Hygroscopicity study of six RA-XII forms at 25°C and 90% RH....8
Figure S12. Characteristic PXRD patterns of RA-XII gram-scaled Form I....9
Figure S13. Calculated and experimental PXRD patterns of Form I....9
Figure S14. Calculated and experimental PXRD patterns of Form II....10
Figure S15. Calculated and experimental PXRD patterns of Form V 10
Figure S16. Characteristic PXRD patterns of RA-XII Form I after drying and before drying....11
Figure S17. Characterization of RA-XII Form I after drying 11
Figure S18. HPLC chromatograms of hydrate (Form I) after measured variable temperature PXRD profile....12
Table S1. Preparation methods of various forms of RA-XII....13
Table S2. The 2-Theta in PXRD patterns of RA-XII forms....14
Table S3. Purity results of stability pre-experiments of three RA-XII solvatomorphs 15
Table S5. The data of equilibrium solubility of six RA-XII forms 15
Table S6. Dissolution curves and fitting equations of 6 API forms in water and pH 7.4 PBS....16
Table S7. Crystal structure parameters of three RA-XII single-crystals....17
Table S8. Hydrogen bonds in crystal structures of Conformation I (Form I) 18
Table S9. Hydrogen bonds in crystal structures of Conformation II (Form II)....19
Table S10. Hydrogen bonds in crystal structures of Conformation III (Form V) 20
Table S11. Summary of the lattice energies 21

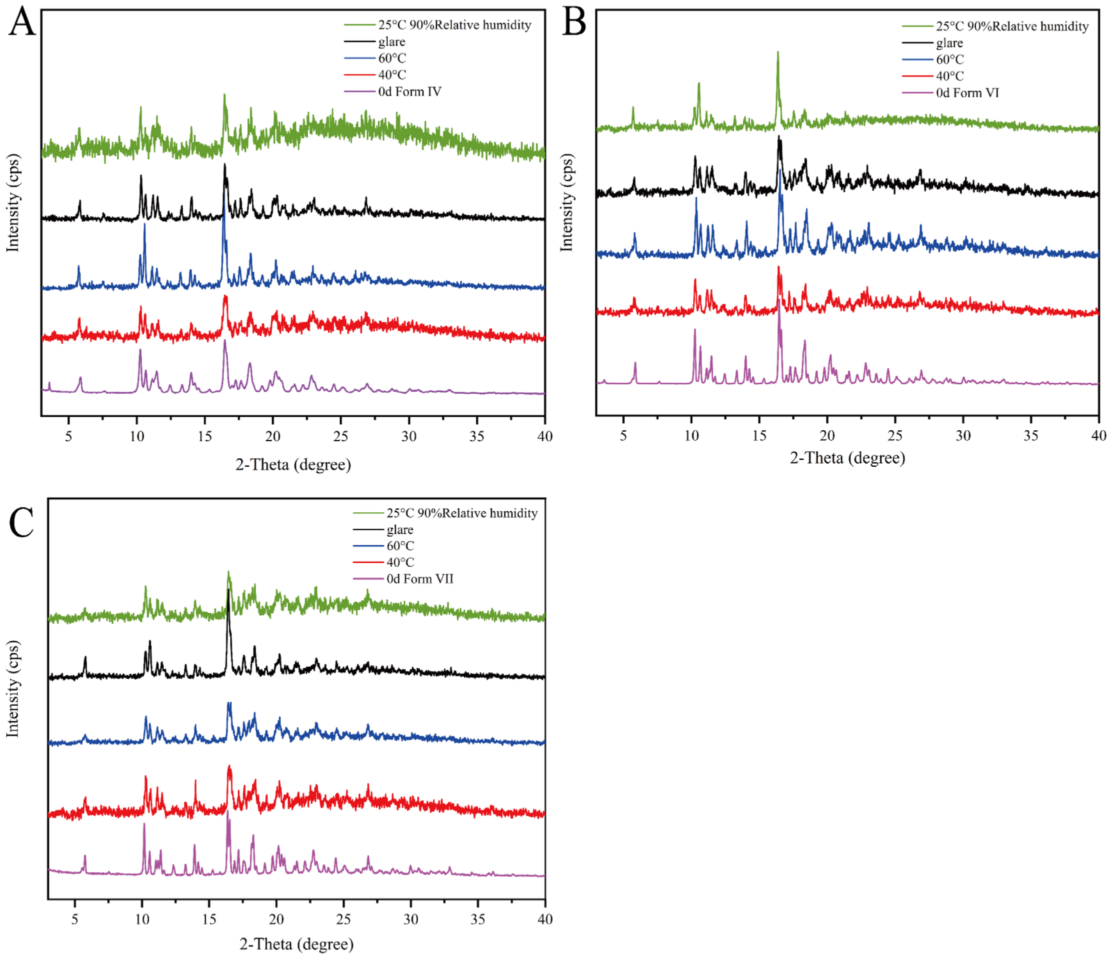

Figure S1. PXRD results of stability pre-experiments of three forms for 10 days (A. Form IV, B. Form VI, C. Form VII)

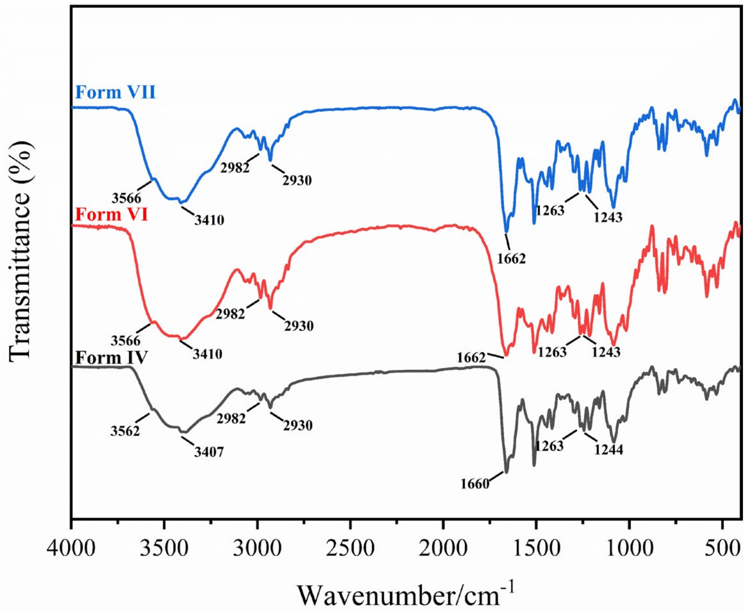

Figure S2. FT-IR spectra of Form IV, VI and VII

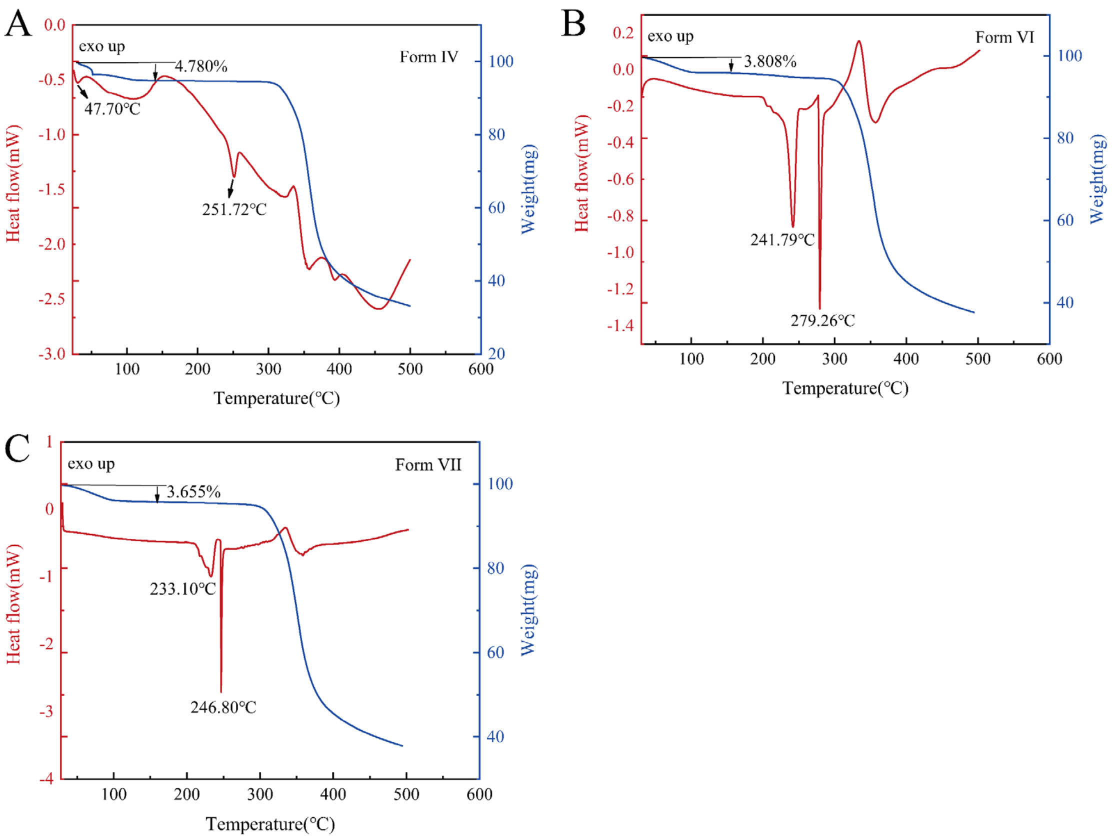

Figure S3. Characterization of three RA-XII solvatomorphs. TGA (blue lines) and DSC (red lines) analyses for (A) Form IV, (B) Form VI, (C) Form VII

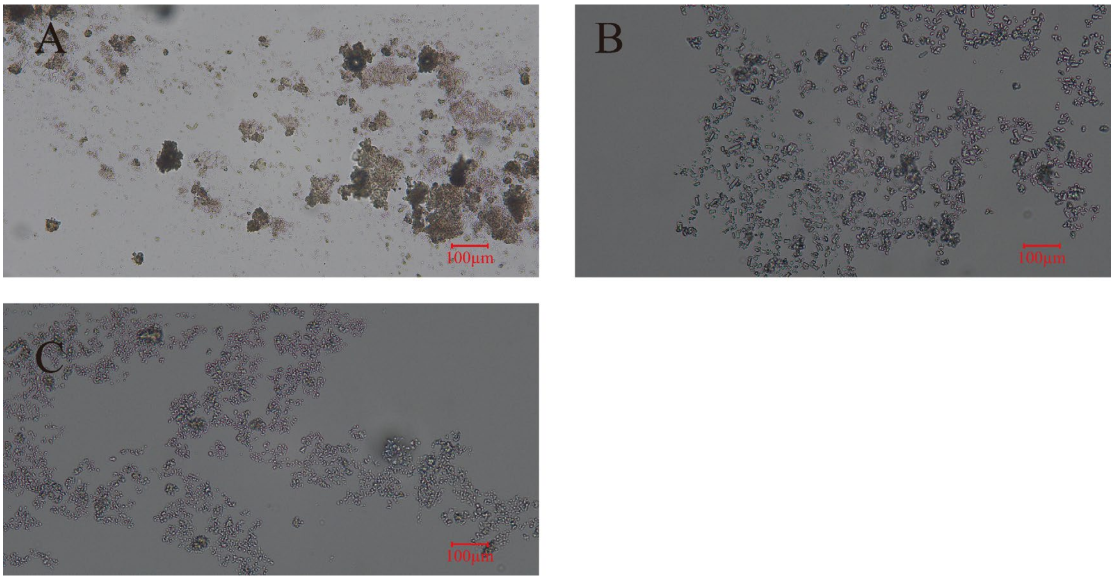

Figure S4. Microscopy images of (A) Form IV, (B) Form VI, (C) Form VII

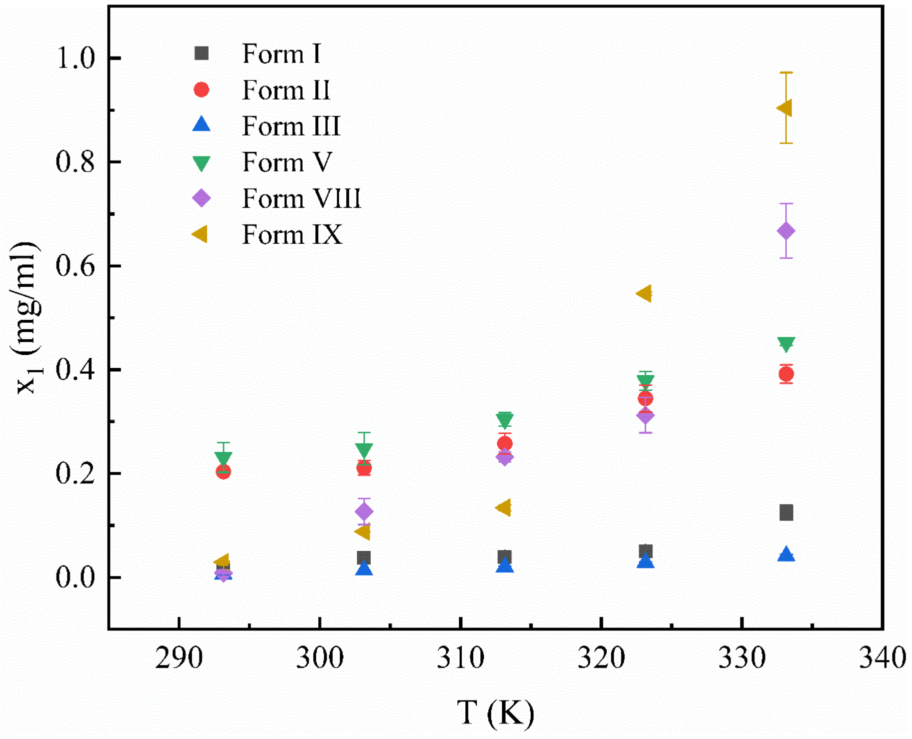

Figure S5. Concentration solubility of six RA-XII forms in water from dynamics of crystallization

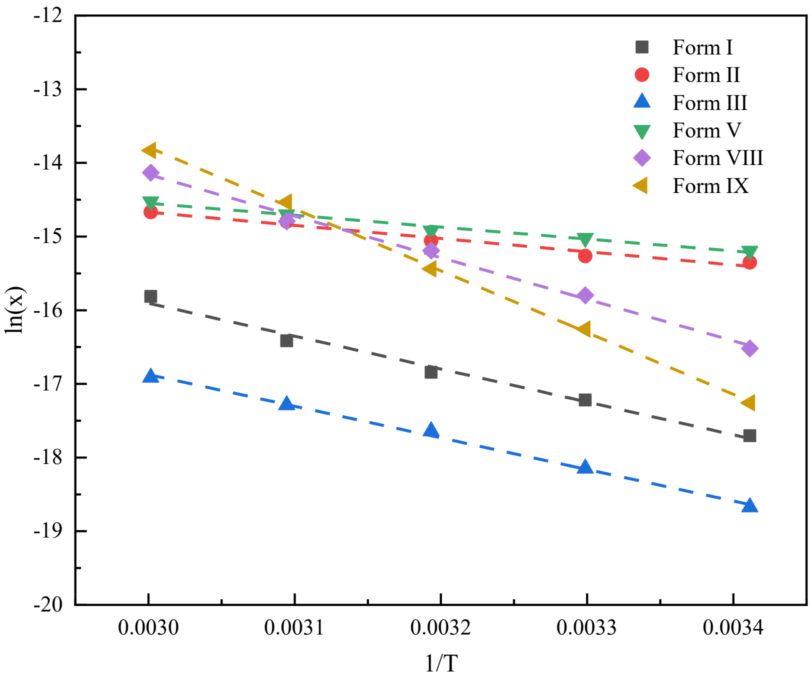

Figure S6. The linear relationship between ln(x) and 1/T for 6 API forms in an aqueous system

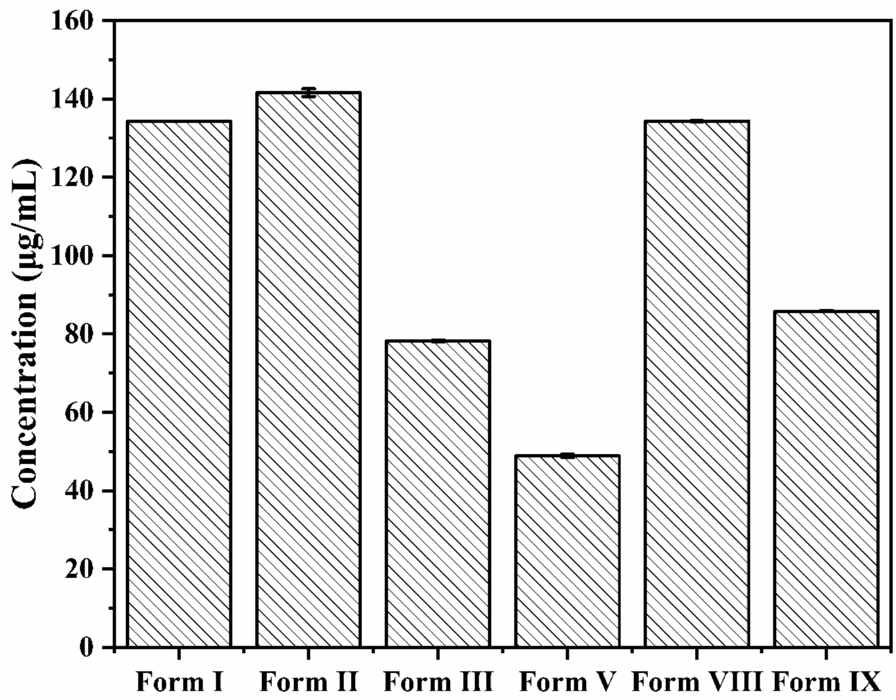

Figure S7. Equilibrium solubility of six RA-XII forms

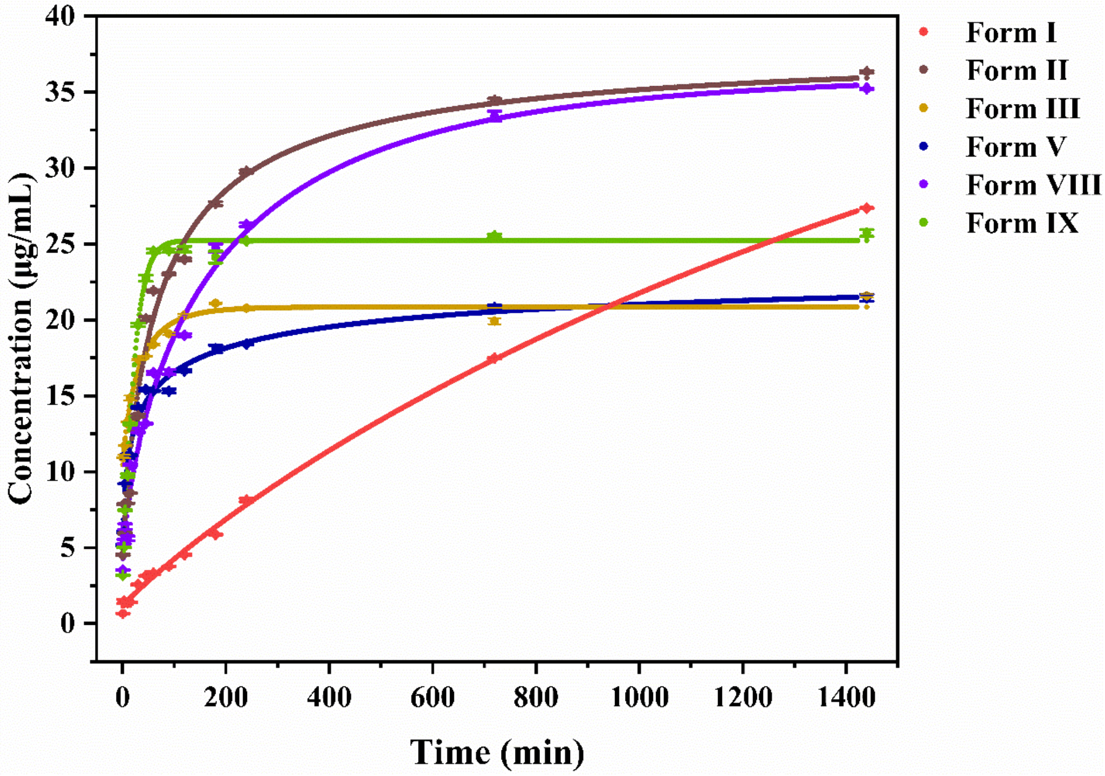

Figure S8. Dissolution kinetics curves of six RA-XII forms in distilled water

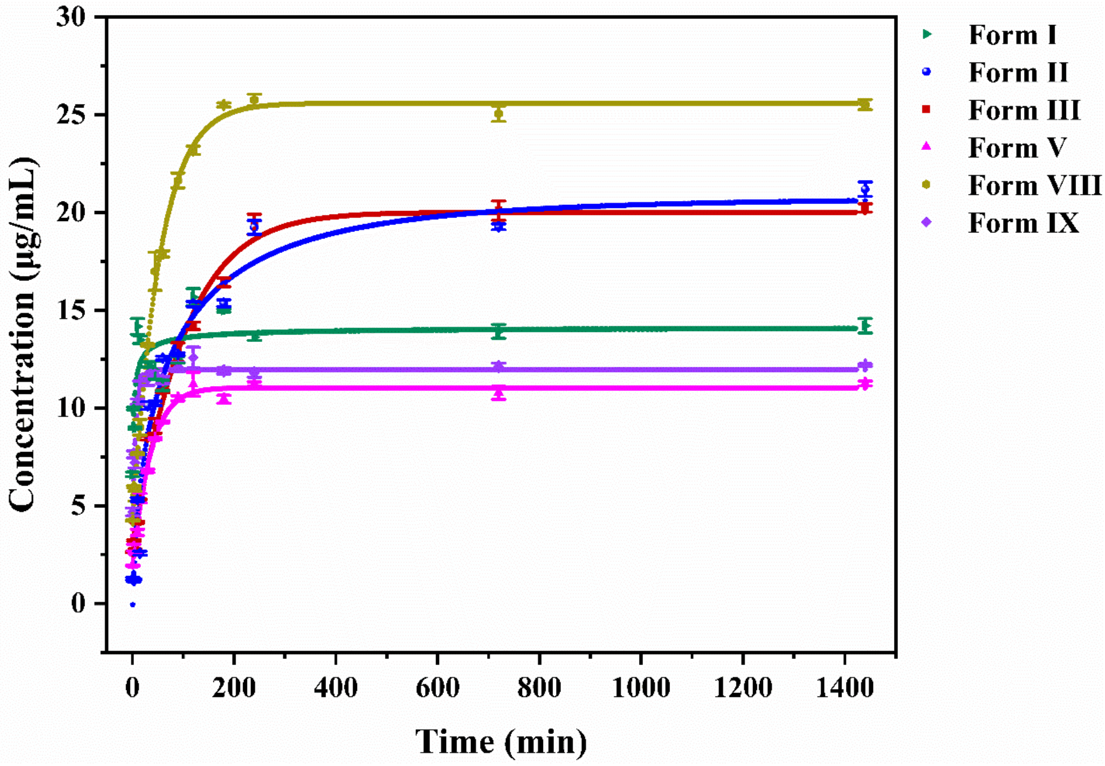

Figure S9. Dissolution kinetics curves of six RA-XII forms in pH 7.4 PBS solution

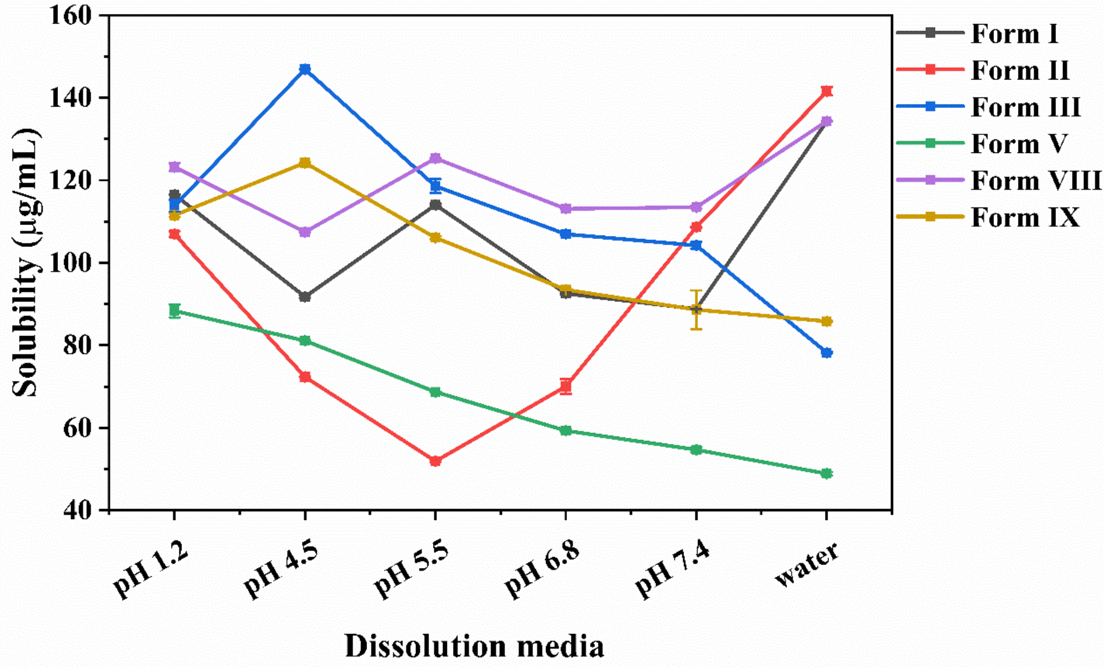

Figure S10. Solubility of six RA-XII forms in different pH buffer solutions

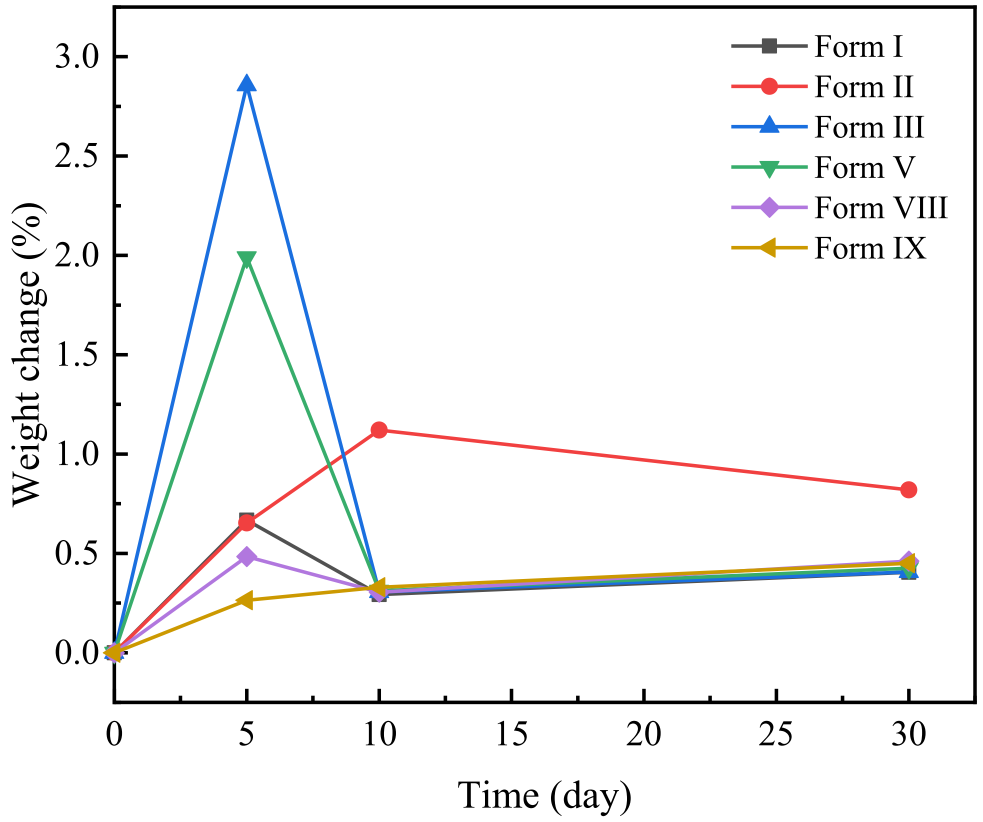

Figure S11. Hygroscopicity study of six RA-XII forms at 25°C and 90% RH

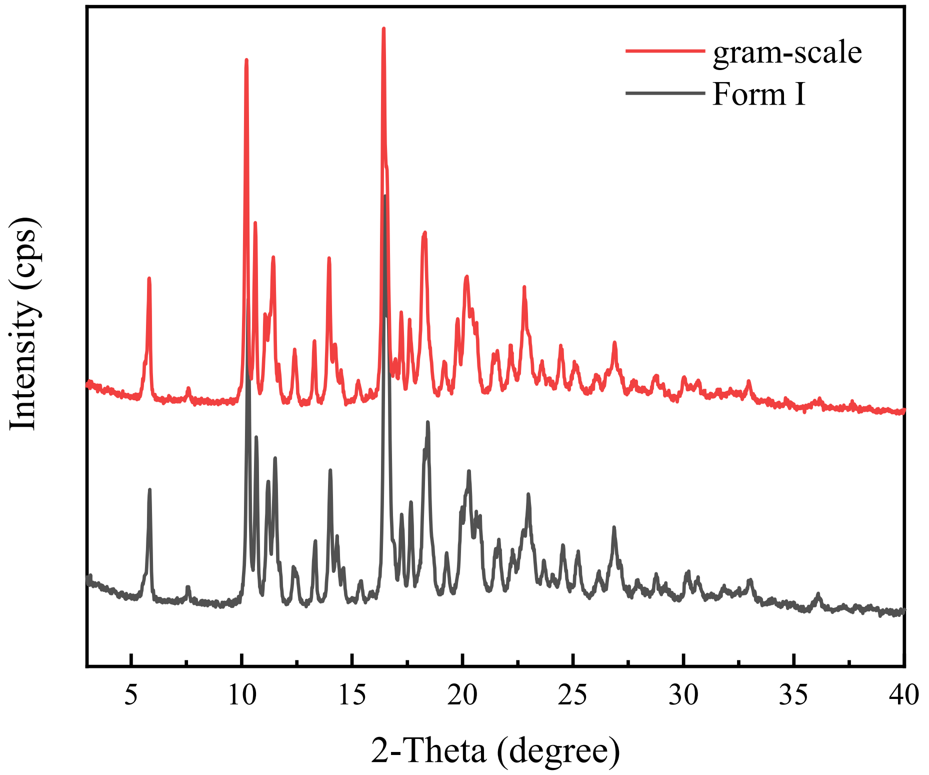

Figure S12. Characteristic PXRD patterns of RA-XII gram-scaled Form I

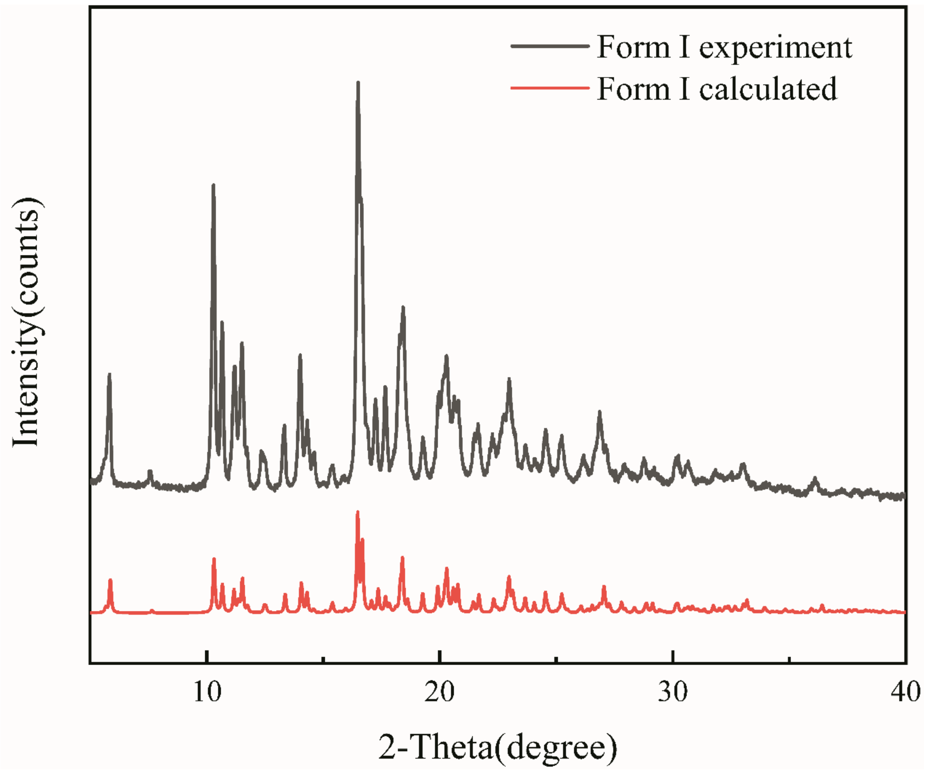

Figure S13. Calculated and experimental PXRD patterns of Form I

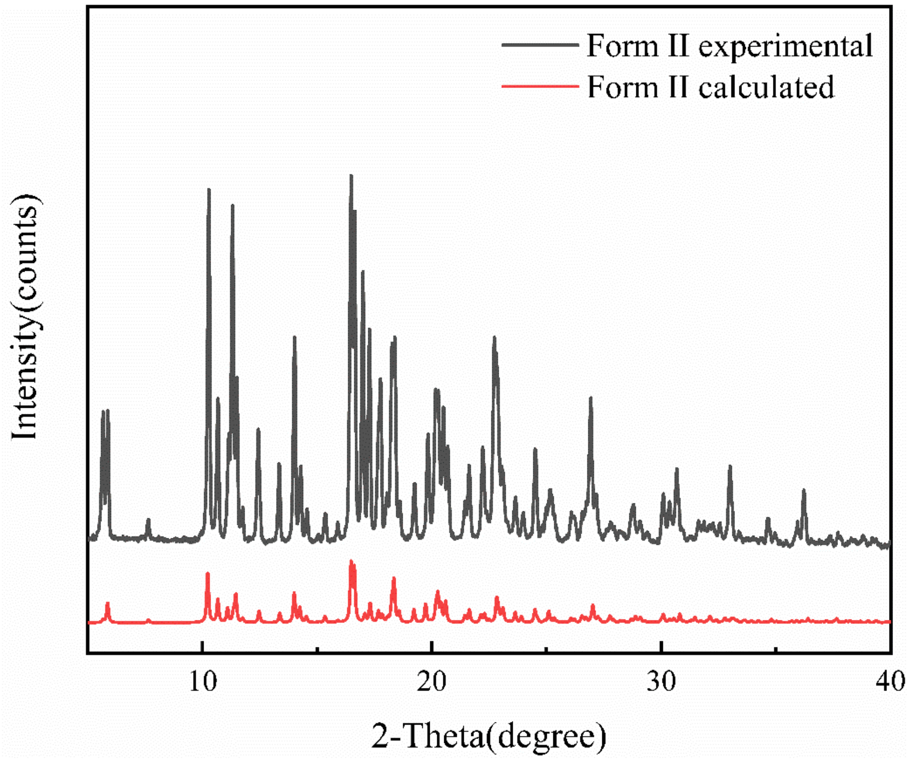

Figure S14. Calculated and experimental PXRD patterns of Form II

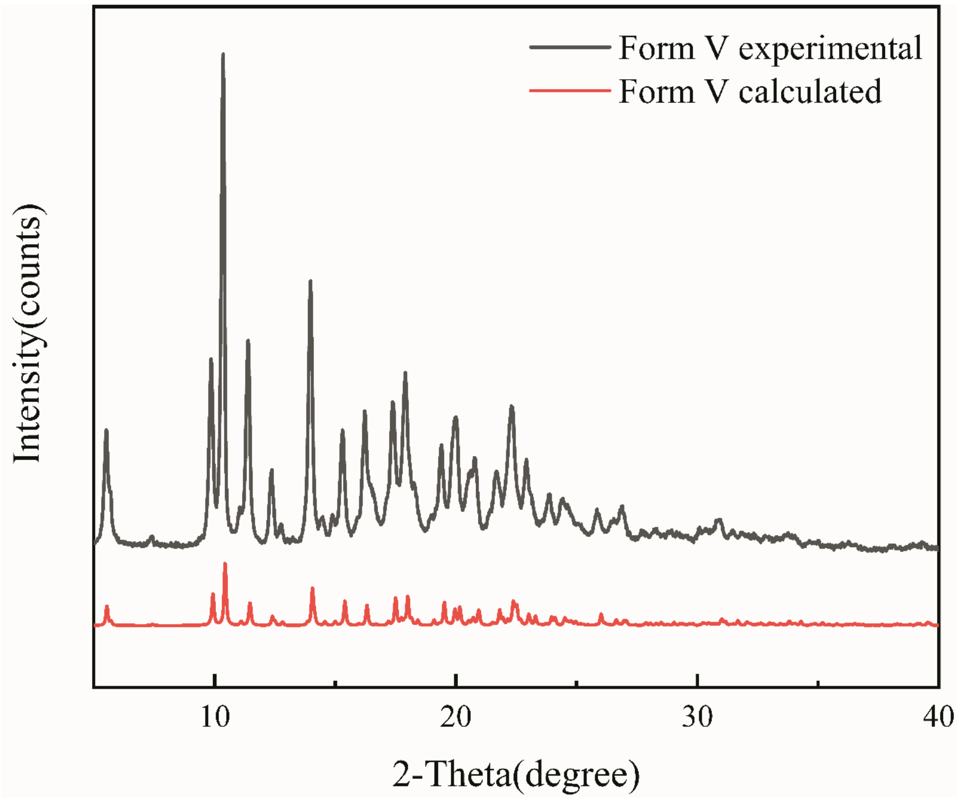

Figure S15. Calculated and experimental PXRD patterns of Form V

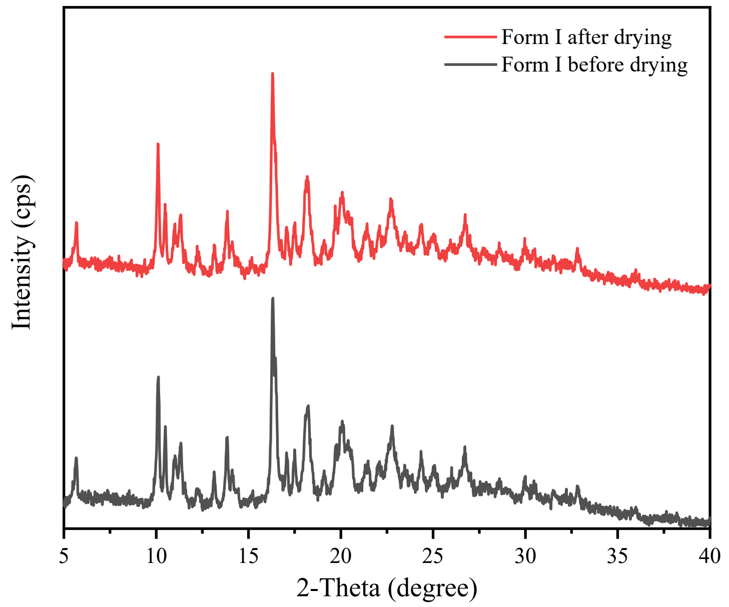

Figure S16. Characteristic PXRD patterns of RA-XII Form I after drying and before drying

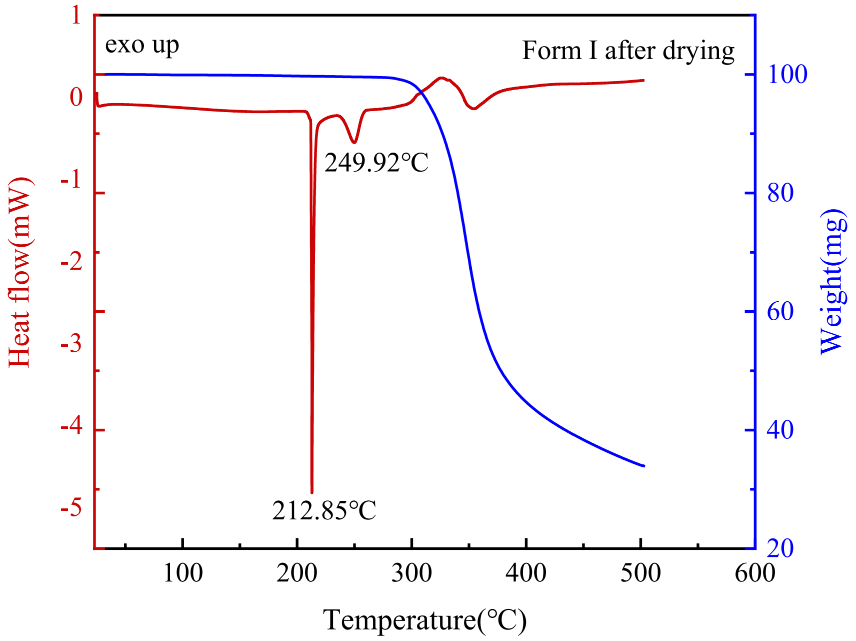

Figure S17. Characterization of RA-XII Form I after drying. TGA (blue lines) and DSC (red lines) analyses,

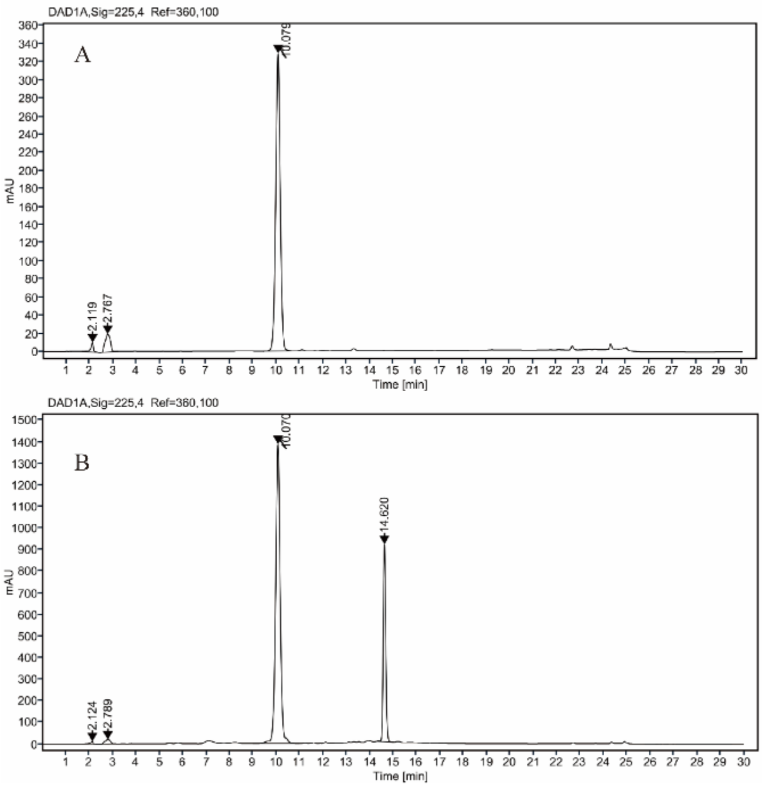

Figure S18. HPLC chromatograms of Form I after measured variable temperature PXRD profile. (A) HPLC analysis of Form I after holding at 50–120°C for 5 min; (B) HPLC analysis of Form I after holding at 50–200°C for 10 min (RA-XII retention time: 10.07 min; RA-V retention time: 14.62 min)

Table S1. Preparation methods of various forms of RA-XII

Forms	solvent and process
Form I	30% ethanol-water, evaporation crystallization, reduce pressure
Form II	1:2 ethyl acetate: water, liquid precipitate crystallization
Form III	9:1 chloroform: methanol, evaporation crystallization, rt
Form IV	petroleum ether, evaporation crystallization, rt
Form V	ethyl acetate, crystal slurry, tem: 40 ℃
Form VI	acetonitrile, evaporation crystallization, rt
Form VII	50% ethanol-water, cooling crystallization, cool rate: 1 ℃/min, blast drying at 70 ℃, atmospheric pressure
Form VIII	50% ethanol-water, cooling crystallization, cool rate: 0.5 ℃/min, blast drying at 150 ℃, at -0.8 mbar
Form IX	50% ethanol-water, cooling crystallization, cool rate: 1 ℃/min, blast drying at 50 ℃, atmospheric pressure

Table S2. The 2-Theta in PXRD patterns of RA-XII forms

Forms	2-Theta (degree)
Form I	**5.84**、10.30、10.67、**11.22**、11.53、13.31、14.01、14.32、**16.51**、17.23、17.65、19.25
Form II	**5.68**、**5.88**、10.29、10.69、11.16、**11.32**、11.50、11.75、12.45、13.35、14.03、14.29、14.57、15.35、15.90、16.48、16.63、**17.00**、17.28、17.66、18.25、18.38、19.25、**19.84**、20.51、20.70、21.63、22.22、23.65、23.98、24.79、26.94、30.09、30.35、30.89、33.01
Form III	Amorphism
Form IV	3.58、5.88、10.26、10.66、11.14、11.46、12.46、13.34、14.00、16.48、17.28、17.66、18.32、19.22、19.80、20.22、21.58、22.20、22.84、24.50、26.90
Form V	**5.56**、5.72、9.87、**9.92**、10.35、**11.89**、12.35、**12.80**、13.98、15.34、16.20、**17.35**、17.86、19.36、19.99、21.69、22.30、22.93
Form VIII	**5.76**、5.80、**10.16**、10.64、**11.40**、12.37、13.30、13.98、16.43、16.60、16.95、17.26、**17.72**、20.45、22.18、22.63、22.70、24.93、26.82、30.65、31.57、32.96、34.61、36.17、38.76
Form IX	5.56、**5.80**、7.56、10.20、**10.58**、11.11、11.42、**11.68**、12.31、13.35、13.94、14.22、14.50、15.29、16.39、16.55、16.87、**17.18**、17.61、**19.16**、19.78、21.38、21.54、22.13、23.54、23.90、24.90、25.09、26.84、29.02、30.01、30.25、30.56、33.27、36.12

Table S3. Purity results of stability pre-experiments of three RA-XII solvatomorphs (Forms IV, VI and VII)

Condition	Purity		
	Form IV	Form VI	Form VII
Base form 0 day	99.54 ± 0.08	99.74 ± 0.07	99.84 ± 0.03
40 °C for 10 days	98.26 ± 0.16	98.83 ± 0.05	98.21 ± 0.03
60 °C for 10 days	95.97 ± 0.50	96.20 ± 0.12	97.21 ± 0.59
Galre for 10 days	97.78 ± 0.36	97.88 ± 0.56	97.05 ± 0.50
25 °C 90%RH for 10 days	97.50 ± 0.53	97.62 ± 0.05	97.63 ± 0.35

Table S4. The parameters of 6 API forms in water system

Forms	ΔH_d/kJ·mol^{-1}	ΔS_d/J·mol^{-1}·K^{-1}
Form I	37.125	20.826
Form II	14.879	77.287
Form III	35.665	33.260
Form V	13.449	80.574
Form VIII	46.924	23.097
Form IX	69.694	94.485

Table S5. The data of equilibrium solubility of six RA-XII forms

Forms	Equilibrium solubility/μg/mL
Form I	134.26 ± 0.06
Form II	141.59 ± 0.96
Form III	78.21 ± 0.20
Form V	48.88 ± 0.39
Form VIII	134.29 ± 0.14
Form IX	85.80 ± 0.16

Table S6. Dissolution curves and fitting equations of 6 API forms in water and pH 7.4 PBS

Forms	Water			pH 7.4 PBS		
	R^2	Dissolution (24 h/μg/mL)	Time to steady-state/min	R^2	Dissolution (24 h/μg/mL)	Time to steady-state/min
Form I	0.9981	27.36	-	0.9972	14.20	8
Form II	0.9957	36.34	50	0.9981	21.29	180
Form III	0.9911	21.58	90	0.9910	20.23	240
Form V	0.9991	21.45	180	0.9946	11.25	90
Form VIII	0.9983	35.21	60	0.9979	25.51	100
Form IX	0.9971	26.73	45	0.9975	12.19	10

Table S7. Crystal structure parameters of three RA-XII single-crystals

Forms	Form I*	Form II*	Form V
Empirical formula	$C_{46}H_{58}N_6O_{14}$, 3(H_2O)	$C_{46}H_{53.77}N_6$ $O_{13.77}$, 2.773(H_2O), 0.227(HO), $H_{0.46}$ $O_{0.23}$	$C_{46}H_{58}N_6O_{14}$, $C_4H_8O_2$
Formula wt	973.02	969.00	1007.09
Cryst syst	Orthorhombic	Orthorhombic	Orthorhombic
Space group (No.)	$P_{21\ 21\ 21}$	$P_{21\ 21\ 21}$	$P_{21\ 21\ 21}$
T(K)	193	193	150
a(Å)	8.9098	8.9927	8.8004
b(Å)	17.1808	17.1818	18.5755
c(Å)	31.1353	31.1265	30.8952
α(deg)	90	90	90
β(deg)	90	90	90
γ(deg)	90	90	90
Z	4	4	4
V(Å^3)	4766.1	4809.4	5050.5
D_{calcd}(g/cm^3)	1.356	1.338	1.324
μ(mm^{-1})	0.871	0.863	0.827
Crystal size/mm^3	0.13×0.12×0.1	0.13×0.12×0.11	0.06×0.05×0.04
H,k,lmax	10,20,37	10,20,37	10,22,37
F000	2072.0	2056.0	2144.0
F000’	2079.12	2063.10	2151.18
no. of rflns collected	8428[4735]	8505[4776]	9802[5480]
no. of params	731	748	693
R1	0.0821(6679)	0.0749(6688)	0.0679(8252)
wR2	0.1945(8282)	0.1918(8496)	0.1639(9708)
S	1.077	1.060	1.126
CCDC No.	2529513	2529518	2529516

“*” Elevated R-factors for Form I and II is primarily from the disordered and partially occupied water molecules and the disorder of the terminal glucoses; both the molecular backbone and the hydrogen-bonding structural units are clearly discernible.

Table S8. Hydrogen bonds in crystal structures of Conformation I (Form I)

Donor --- H....Acceptor			D - H	H...A	D...A	D - H...A
<u>N2</u>	<u>H2</u>	<u>O9</u>	0.88	2.03	2.875(6)	160
N3	H3	O5	0.88	2.27	2.633(4)	105
N3	H3	N1	0.88	2.48	2.775(4)	101
N4	H4A	O44A	0.88	2.36	3.176(11)	153
<u>O8</u>	<u>H8C</u>	<u>O6</u>	0.87	2.01	2.873(7)	176
<u>O9</u>	<u>H9A</u>	<u>O2</u>	0.87	2.05	2.802(7)	144
O14	H14	O7	0.84	2.31	3.077(10)	152
O14	H14	O8A	0.84	2.40	2.994(14)	128
<u>O44A</u>	<u>H44D</u>	<u>O4</u>	0.84	2.15	2.823(10)	137
O47A	H47A	O8A	0.84	2.51	2.870(16)	107
O47A	H47A	O14	0.84	2.17	2.857(14)	139
<u>O48A</u>	<u>H48A</u>	<u>O8</u>	0.84	1.93	2.578(11)	133
C8	H8A	O3	0.99	2.50	2.923(5)	106
C12	H12	O15	1.00	2.29	3.077(4)	135
C15	H15	O	1.00	2.43	2.810(6)	102
C22	H22	O5	1.00	2.26	2.716(4)	106
C33	H33	N6	0.95	2.59	3.102(5)	114
C36	H36A	O6	0.98	2.58	3.089(6)	113
C36	H36B	O15	0.98	2.25	2.657(6)	104

Table S9. Hydrogen bonds in crystal structures of Conformation II (Form II)

Donor --- H....Acceptor			D - H	H...A	D...A	D - H...A
N3	H3B	O15	0.88	2.23	3.064(12)	159
O10	H10	O16	0.84	1.94	2.695(9)	148
O10	H10A	O14A	0.87	2.10	2.739(12)	129
O11	H11A	O6	0.84	1.98	2.735(8)	149
O13	H13	O16	0.84	2.12	2.625(11)	118
O13A	H13A	O4	0.98	2.50	3.284(10)	137
O14	H14B	O15	0.84	2.21	2.739(12)	121
O16	H16A	O13	0.87	1.98	2.625(11)	130
O16	H16B	O17A	0.87	2.38	2.860(4)	116
O16A	H16C	O3	0.89	1.92	2.400(3)	112
O16A	H16D	O8	0.87	2.19	2.940(2)	144
O17	H17C	O11A	0.87	2.01	2.590(3)	123
O17	H17C	O17A	0.87	1.64	2.360(3)	137
O17	H17D	O5	0.87	2.53	2.895(14)	107
C6	H6	O14	1.00	2.52	2.885(12)	101
C8	H8	O12	0.95	2.54	2.919(14)	104
C11	H11	N6	0.95	2.58	3.090(8)	114
C17	H17	O6	1.00	2.26	2.715(7)	106
C25	H25A	O17A	0.98	2.05	2.870(4)	140
C28	H28	O5	1.00	2.41	2.787(10)	102
C29	H29C	O17A	0.98	2.16	3.000(3)	142
C35	H35	O7	1.00	2.32	3.119(8)	136
C39	H39A	O2	0.99	2.48	2.917(9)	106

Table S10. Hydrogen bonds in crystal structures of Conformation III (Form V)

Donor ---	H....	Acceptor	D - H	H...A	D...A	D - H...A
N3	H3	N18	0.79(6)	2.38(5)	2.767(5)	111(4)
N12	H12	O50	0.71(4)	2.29(4)	2.946(7)	156(4)
N15	H15	O67	0.88	2.10	2.888(5)	148
O50	H50	O60	0.84	2.33	3.110(3)	154
O51B	51B	O52	0.84	2.46	2.790(3)	104
O52	H52	O58	0.84	2.17	2.990(5)	164
O54	H54	O59	0.84	2.17	2.722(12)	123
O54A	54A	O51A	1.24	2.46	3.480(6)	138
C7	H7	O58	1.00	2.25	2.719(6)	107
C13	H13	O57	1.00	2.42	2.816(6)	103
C13	H13	O67	1.00	2.51	3.264(6)	132
C16	H16	O56	1.00	2.41	3.243(5)	140
C19	H19A	O55	0.99	2.54	2.990(7)	108
C40	H40	O44	0.95	2.59	2.933(6)	102
C46	H46A	O69	1.00	2.58	3.540(7)	160
C62	H62C	O56	0.98	2.36	3.264(6)	153
C63	H63A	O56	0.98	2.30	2.649(7)	100
C66	H66A	O54	0.98	2.39	3.285(13)	152
C70	H70A	O57	0.99	2.31	3.213(7)	151
C71	H71B	O52	0.98	2.55	3.487(8)	160

Table S11. Summary of the lattice energies

Solvatomorphs	Lattice energy (kJ/mol)
Form I	-221.44
Form II	-215.27
Form V	-207.65
